# In-depth genome and pan-genome analysis of a metal-resistant bacterium *Pseudomonas parafulva* OS-1

**DOI:** 10.3389/fmicb.2023.1140249

**Published:** 2023-06-20

**Authors:** Kiran Kumari, Vaishnavi Rawat, Afreen Shadan, Parva Kumar Sharma, Sushanta Deb, Rajnish Prakash Singh

**Affiliations:** ^1^Department of Bioengineering and Biotechnology, Birla Institute of Technology, Ranchi, Jharkhand, India; ^2^Department of Microbiology, Dr. Shyama Prasad Mukerjee University, Ranchi, India; ^3^Department of Plant Sciences and Landscape Architecture, University of Maryland, College Park, MD, United States; ^4^Department of Veterinary Microbiology and Pathology, Washington State University (WSU), Pullman, WA, United States

**Keywords:** carbohydrate active enzymes, genome, *Pseudomonas parafulva*, pan-genome, virulence

## Abstract

A metal-resistant bacterium *Pseudomonas parafulva* OS-1 was isolated from waste-contaminated soil in Ranchi City, India. The isolated strain OS-1 showed its growth at 25–45°C, pH 5.0–9.0, and in the presence of ZnSO_4_ (upto 5 mM). Phylogenetic analysis based on 16S rRNA gene sequences revealed that strain OS-1 belonged to the genus *Pseudomonas* and was most closely related to *parafulva* species. To unravel the genomic features, we sequenced the complete genome of *P. parafulva* OS-1 using Illumina HiSeq 4,000 sequencing platform. The results of average nucleotide identity (ANI) analysis indicated the closest similarity of OS-1 to *P. parafulva* PRS09-11288 and *P. parafulva* DTSP2. The metabolic potential of *P. parafulva* OS-1 based on Clusters of Othologous Genes (COG) and Kyoto Encyclopedia of Genes and Genomes (KEGG) indicated a high number of genes related to stress protection, metal resistance, and multiple drug-efflux, etc., which is relatively rare in *P. parafulva* strains. Compared with other *parafulva* strains, *P. parafulva* OS-1 was found to have the unique β-lactam resistance and type VI secretion system (T6SS) gene. Additionally, its genomes encode various CAZymes such as glycoside hydrolases and other genes associated with lignocellulose breakdown, suggesting that strain OS-1 have strong biomass degradation potential. The presence of genomic complexity in the OS-1 genome indicates that horizontal gene transfer (HGT) might happen during evolution. Therefore, genomic and comparative genome analysis of *parafulva* strains is valuable for further understanding the mechanism of resistance to metal stress and opens a perspective to exploit a newly isolated bacterium for biotechnological applications.

## Introduction

The contamination of the environment with heavy metals and other pollutants possesses a serious threat to human health. Additionally, the industrial release of extensive metals and chemicals has resulted in the accumulation of a higher amount of effluents containing toxic heavy metals, and these effluents create environmental disposal problems due to their non-degradable and persistent characteristics (Tchounwou et al., 2013). Initially, heavy metals show high toxicity against most microorganisms (Lemire et al., 2013), however, microorganisms including bacteria have evolved several molecular mechanisms such as detoxification, bioprecipitation, and bioaccumulation to cope with heavy metal toxicity (Lemire et al., 2013). Therefore, understanding the mechanisms of metal resistance and identification of new bacterial strain is crucially important, and imperative to remove and recover heavy metals from polluted environments. To better understand the genetic architecture providing support to the growth of a microorganism under abiotic stressors, here, we investigated the genomic features of a newly isolated metal-resistant bacterium *P. parafulva* OS-1.

The genus *Pseudomonas* represents diverse groups of microorganisms capable to survive under various stressors including multi-metal pollution (Zhang et al., 2012; Moreno and Rojo, 2013). Members of *Pseudomonas* are well capable to colonize different kinds of environments and also possess the strong ability to degrade toxic organic pollutants (Wu et al., 2011). Das et al. (2015) suggested that abundant genetic diversity of *Pseudomonas* spp. supports their survival under diverse environmental conditions. Meanwhile increasing research has revealed the potential of *Pseudomonas* strains for their ability to remediate polluted areas, which supports sustainable agriculture production and environmental protection (Bello-Akinosho et al., 2016; Das et al., 2020). A previous study showed that the bacterium *Pseudomonas* sp. Ph6 was able to degrade the phenanthrene (Sun et al., 2014), whereas *Pseudomonas* sp. P3 showed its efficacy against pyrenes, naphthalene, fluorine and other organic compounds (Zhu et al., 2016). A biosurfactant-producing bacterium *Pseudomonas aeruginosa* L10 was able to degrade the crude oil (Wu et al., 2018). *P. plecoglossicida* 2.4-D and *P. hunanensis* IB C7 were found to be efficient for high oil degradation (70%) and also maintained plant growth in petroleum pollutants (Bakaeva et al., 2020). *P. putida* VM1450 showed its potential for clean-up of soil contaminated with 2,4-dichlorophenoxyacetic acid, thereby enhancing the removal of herbicide from the soil (Germaine et al., 2006). Some of the rhizosphere *Pseudomonas* strains showed resistance to heavy metals and also possess various features, which make them a potent tool for the remediation of heavy-metal pollution (Płociniczak et al., 2013; Chellaiah, 2018). These metal-resistant bacteria also contribute to the mobilization and uptake of heavy metals, thereby improving plant growth under metal stressors (Płociniczak et al., 2019). *P. helmanticensis* strain H16 treated *Silene vulgaris* shoot tissue showed an increase in Zn and Cd accumulation by 43.8 and 112.6%, respectively (Płociniczak et al., 2019).

Recently, the development in sequencing technologies and comparative genomics has been exploited to explore the many secondary metabolite pathways in the microbes, which contribute to its potential and environment suitability (Dickschat, 2016). The presence of secondary metabolites biosynthetic gene clusters (BGCs) such as non-ribosomal peptide synthesis (NRPS), polyketide synthesis (PK), and other secondary metabolite-producing genes in the genome of microorganisms equipped them for a variety of agricultural, pharmaceutical, and industrial applications (Dastager et al., 2015; Yamada et al., 2015; Meng et al., 2020). These widely distributed secondary metabolite gene clusters are necessary for the growth, adaptation to the environment and defense mechanism, but sometimes also confer pathogenicity and virulence (Wang and Lu, 2017). Similarly, the production of various antibiotic metabolites, lipopeptides, and biofilm formation contributes to its biocontrol capabilities by creating a mechanical barrier between the pathogen and nutrient site (Wallace et al., 2018). In terms of biocontrol action, a study showed that *P. parafulva* inhibited the growth of various bacterial and fungal pathogens (Uchino et al., 2001). Pyrimidine and benzoate-producing *P. parafulva* strain CRS01-1 showed antagonistic activity against *P. fuscovaginae*, *Acidovorax avenae*, and *Rhizoctonia soalni* etc. (Liu et al., 2015). Various metabolite gene clusters associated with pyrimidine and benzoate synthesis used to control the pathogenic fungi, and bacteria were identified in *P. parafulva* CRS01-1 (Liu et al., 2015). Similarly, phenazine-1-carboxylic acid-producing bacterium *P. parafulva* JBCS1880 inhibited the proliferation of *P. cichorii*, *Xanthomonas glycines*, *R. solani*, and *B. glumae* (Zhang Y. et al., 2018; Kakemboa and Lee, 2019).

The use of high-throughput sequencing and advanced genomics may explore a deep understanding of the genetic basis of metabolic potential and environmental adaption (Chen et al., 2018). In the present study, *P. parafulva* OS-1 strain isolated from soils contaminated with heavy metals and other pollutants was investigated. The whole-genome shotgun (WGS) sequencing was done to unravel the genes necessary for stress protection, functional potential, and other beneficial traits. Comparative genomics was performed by evaluating its proximity to related strains and by comparing its whole genome to 10 closely related strains in terms of the core and pan-genomes, which offer insights into evolutionary changes between *parafulva* strains. To the best of our knowledge, the present study is the first report on the in-depth genome and pan-genome analysis of a heavy-metal-resistant bacterium belonging to *P. parafulva*.

## Materials and methods

### Sampling and bacterial isolation

The soil sample was collected from the waste site Jhiri, Ranchi (23.40° N, 85.25° E) in June 2022 and immediately brought to the laboratory in ziplock bags. Two grams of soil was suspended in 18 mL of 0.9% NaCl (w/v) in a 150 mL Erlenmeyer flask and suspension was incubated at 28°C for 2 h on a rotary shaker at 180 rpm. Serial dilution of the suspension was prepared and plated on the LB-agar (Himedia, India) plate supplemented with different concentrations (1 to 5 mM) of heavy metal such as zinc sulfate (ZnSO_4_), copper sulfate (CuSO_4_), cadmium chloride (CdCl_2_), mercuric chloride (HgCl_2_) and nickel sulfate hexahydrate (NiSO_4_.6H_2_O), and incubated at 37°C for 24 to 48 h. One colony showing the optimum growth on the ZnSO_4_-amended (up to 5 mM) LB-agar plate was labeled as OS-1 and was used for further detailed characterization. To determine the growth pattern of the test isolate, one loop of a single colony was inoculated in 100 mL of LB-broth medium supplemented with ZnSO_4_ (1 to 5 mM), as well as other tested heavy metals, and grown in a shaker incubator at 37°C with 180 rpm for 24 h. The growth pattern was determined by using a spectrophotometer (HACH, United States) after a four-hour interval by measuring optical density (OD) at OD590. The strain was maintained in 20% glycerol in a − 80°C freezer (Thermo Fisher Scientific, United States).

### Molecular identification

A single colony of OS-1 was inoculated into 5nml of LB-broth for isolation of genomic DNA. Genomic DNA was extracted using the Qiagen DNA isolation kit (Qiagen, Germany) and the purity of extracted DNA was checked using NanoDrop 2000 UV–Vis spectrophotometer (Thermo Scientific, United States). For 16S rRNA amplification, 200 ng of genomic DNA was amplified using universal primers 27F1 (AGAGTTTGATCCTGGCTCAG) and 1492R (TACGGCTACCTTGTTACGAC) (Lane, 1991) in 18 μL of PCR mixture (2X Taq Polymerase Master Mix, Takara Life Sciences) with 0.75 mM of each primer set in a Thermocycler (Biorad, United States). The condition involved an initial denaturation at 95°C for 5 min followed by 30 cycles with steps of 94°C for 30 s, 55°C for 45 s, and 72°C for 1 min 30 s, and a final extension of 10 min at 72°C. The 16S rRNA sequencing was performed at Eurofins Genomics Ltd. (Eurofins, Bangalore, India) and taxonomic affiliation was assigned using the RDP database^1^ at 98% threshold. The obtained sequence was compared against the GenBank database using the NCBI BLAST algorithm^2^ and deposited in the NCBI database. The phylogenetic tree was constructed using the 16S rRNA sequence of strain OS-1 and other *Pseudomonas* strains following the Neighbor-Joining (NJ) method with 1000 bootstraps replicates using the MEGA 7.0 program (Tamura et al., 2021).

### Biochemical characterization

Gram staining was performed with a Gram-staining kit (Himedia, India). Test for starch hydrolysis, IMViC (Indole, Methyl Red, Voges Proskauer, Citrate utilization), cellulase, pectinase, and lipase was performed following the standard protocol (Harley and Prescott, 2002). Catalase and oxidase activity was performed by using 3% (v/v) H_2_O_2_ and using an oxidase reagent (BioMerieux, France), respectively. The test of growth at different temperatures was done between 20°C to 45°C in sterile LB-medium. pH of LB-medium was adjusted with suitable buffers and tolerance to different pH (5.0 to 9.0) was examined. The ability to utilize different carbon sources was tested by the Himedia Carbohydrate utilization kit (KB009). The selected isolate was tested for its susceptibility against different antibiotics following CLSI (Clinical and Laboratory Standards Institute) instruction. The freshly grown culture of OS-1 was spread on an LB-agar plate and a paper disk containing different antibiotics namely ampicillin, kanamycin, erythromycin, tetracycline, ciprofloxacin, gentamicin, and streptomycin were placed, and incubated for 24–48 h. The result was interpreted by measuring the zone of inhibition (ZOI) created by the antibiotics disk. The isolate was tested for various motility behaviors such as swimming, swarming, and twitching following standard protocol (Connelly et al., 2004).

### Whole genome sequencing

A single colony of OS-1 was inoculated into 10 mL of LB-broth medium and incubated at 37°C on a rotary shaker at 180 rpm overnight. Genomic DNA was extracted using the Qiagen DNA isolation kit and sequenced using an Illumina MiSeq platform, and the paired-end library was prepared by using the NEB Next Ultra DNA Library Prep Kit. Fast QC program was used for quality control of Illumina reads.^3^ Trimming of raw reads was performed using Trimgalore Version 0.6.7 and Sickle Galaxy version 1.33.2 (Schmieder and Edwards, 2011). This tool generates trimmed forward reverse reads along with a singleton file of the sample OS1. Further, assembly was performed using ABySS Version 2.3.4 (Simpson et al., 2009) with a kmer length of 41 which generated a scaffold file of 199 MB. The Illumina reads were further assembled using genome assembly tools SPAdes (Nurk et al., 2013). The further genome sequence was annotated by Rapid Annotation using Subsystem Technology (RAST) annotation (Aziz et al., 2008). COG functions of protein-coding sequences were determined using the RPS-BLAST algorithm for BLAST search against the COG database (Schaffer et al., 2001; Deb and Das, 2022).^4^ The heat map clustering of the *P. parafulva* strains based on COG functional profile was obtained using the Manhattan distance and average clustering method implemented in the Heatmap-2 function of the Gplots package (Warnes et al., 2016). The genes involved in biological pathways were annotated using KEGG and Blast2Go tools (Kanehisa and Goto, 2000).

### Average nucleotide identity analysis

ANI was performed to explore the genetic distance and relatedness for the genome sets containing OS-1 and publicly available *Pseudomonas* genomes (Table 1) by ANI-BLAST (ANIb) using the software JSpecies v.1.2.1^5^ with a threshold of 95% as the cut-off for species (Richter and Rossello-Mora, 2009). The occurrence of ANIb value equal to or more than 95% represents the strains belonging to the same species (Lalucat et al., 2020). The ANI matrix was visualized using the tool pyani (Pritchard, 2016).

**Table 1:** The genome features of various *P. parafulva* strains.

Strains	Accession	Size [bp]	GC-content [%]	CDS (pseudo)	rRNA	tRNA
*Pseudomonas parafulva* NBRC 16636	NZ_KE384443	4,956,622	62.49	4,468 (66)	5	55
*Pseudomonas parafulva* DSM 17004	NZ_BBIU01000055	4,954,362	62.48	4,466 (62)	5	59
*Pseudomonas parafulva* strain CRS01-1	NZ_CP009747	5,087,619	63.46	4,372 (48)	21	76
*Pseudomonas parafulva* strain DTSP2	NZ_JAEMEE010000001	4,623,712	61.8	4,135 (78)	5	70
*Pseudomonas parafulva* strain JBCS1880	NZ_CP031641	5,208,480	63.38	4,555 (37)	19	75
*Pseudomonas parafulva* strain NS212	NZ_LDSM01000001	4,823,531	61.8	4,370 (88)	6	65
*Pseudomonas parafulva* strain NS96	NZ_LDSN01000001	4,671,094	61.87	4,266 (91)	4	64
*Pseudomonas parafulva* strain PRS09-11288	NZ_CP019952	4,690,783	61.71	4,126 (56)	22	75
*Pseudomonas parafulva* strain PSB00030	NZ_JADTVZ010000001	4,813,337	61.83	4,380 (82)	4	66
*Pseudomonas parafulva* OS-1	NA	5,453,712	62	5,127 (77)	NA	71

### Antimicrobial and virulence analysis

The CARD database was used using a homology-based approach (BLASTX) against the genome sequence of OS-1 to unravel the presence of AMR genes (Alcock et al., 2020). For searching, BLAST output was filtered with a minimum of 80% identity and subject protein coverage. Similarly, the VFDB database was used against assembled genome with criteria of a minimum of 80% identity using a homology-based approach (BLASTX) to identify the virulence genes (Liu et al., 2019).

### Prediction of biosynthetic gene clusters

The number and types of secondary metabolite BGCs in the genome sequence of *P. parafulva* OS-1 were identified by antiSMASH version 5.1.2 in combination with Hidden Markov Model (HMM) to detect the BGCs-like region (Blin et al., 2021). Various unknown and characterized BGCs were identified and genetic similarities in gene clusters were predicted using antiSMASH 5.1.2.

### Prediction of carbohydrate-active enzymes

To reveal the presence of various CAZymes including glycosyltransferases (GTs), glycoside hydrolases (GHs), polysaccharide lyases (PLs), carbohydrateesterases (CEs), auxiliary activities (AAs) and carbohydrate-binding modules (CBMs), the protein sequences of *P. parafulva* OS-1 was annotated using the dbCAN2 server and BLAST-driven DIAMOND against the CAZy database (Zhang H. et al., 2018). The diversity of CAZymes in the closest relatives of *P. parafulva* species (provided in Table 1) was evaluated for the comparative distribution.

### Comparative genome analysis

The analysis of orthologous gene clusters was analyzed using the Orthovenn2 program using protein sequences of *P. parafulva* OS-1, *P. parafulva* PRS09-11288 (NZ_CP019952), *P. parafulva* strain DTSP2 (NZ_JAEMEE010000001), *P. parafulva* strain NS212 (NZ_LDSM01000001), *P. parafulva* strain NS96 (NZ_LDSN01000001), and *P. parafulva* strain PSB00030 (NZ_JADTVZ010000001). These strains were selected as they showed ANI threshold value (> = 96%). The circular genome comparison of the draft assembly genome of OS-1 was performed against the reference genomes using the Blast Ring Image Generator Tool (BRIG) (Alikhan et al., 2011).

### Pan-genome analysis

For pan-genome analysis, the genomes were downloaded from the NCBI database and analyzed using the Get-homologs program to determine the core and pan-genome of *P. parafulva* strains (Contreras-Moreira and Vinuesa, 2013). To estimate core genes, a BlastP search was performed with 1.0 e^−15^ e-value cut-off and ≥ 50% to ≥70% alignment coverage as described in earlier studies (Graña-Miraglia et al., 2018; Das et al., 2021). Subsequently, 75% of sequence identity was applied in BLAST pair wise alignments to demarcate the pan-genomic repertoire of *P. parafulva* species (Bach et al., 2022). The core-genome maximum-likelihood (ML) tree was generated with the help of RAxML tool with a generalized time-reversible (GTR) model (Stamatakis, 2014). The pan-genome matrix file was obtained using scripts compare_clusters.pl. and parse_pangenome_matrix.pl. that were eventually used to construct maximum-likelihood (ML) pan-genome phylogeny (Vinuesa et al., 2018). TreeCmp estimates the dissimilarity in tree topology or branch order of core and pan-genome trees based on normalized matching-cluster (nMC) score, and normalized Robinson-Foulds (nRF) score (Bogdanowicz et al., 2012). Pan-gene classification was performed with the help of an auxiliary script parse_pangenome_matrix.pl. into four major categories; core, soft-core, cloud, and shell (Contreras-Moreira and Vinuesa, 2013). The preliminary concept of pan-genome compartments or categories was proposed by Wolf et al. (2012). The genes of the soft core section were determined following the work of Kaas and collaborators (Kaas et al., 2012), whereas, cloud and shell genes were categorized according to Vernikos et al. (2015).

## Results

### Biochemical characterization

A plate culture image of a newly isolated bacterial strain OS-1 has been provided in Supplementary Figure 1A. The growth kinetics study clearly illustrates that isolate showed the higher growth capacity at 5 mM of ZnSO_4_ stress (Supplementary Figure 1B), as compared to other tested heavy metals (Supplementary Figures 1C–F). The isolate was found to be gram-negative and it showed a negative result for IMViC, amylase, pectinase, and positive for lipase, cellulase, and catalyze test (Supplementary Table S1). Among the tested various carbon sources, the strain was able to utilize lactose, xylose, maltose, galactose, melibiose, sorbitol, glycerol, D-arabinose, citrate, malonate, melezitose, malonate, trehalose, raffinose, ONPG, and mannosidase (Supplementary Table S1). The isolate showed a higher sensitivity (20 to 25 mm) against tetracycline, ciprofloxacin, vancomycin, voriconazole, erythromycin, and moderate sensitivity (12 to 18 mm) to streptomycin, ampicillin, gentamicin, kanamycin, fluconazole (Supplementary Table S2). The isolate showed the optimum growth in pH 6.0 to 9.0. OS-1 showed optimum growth up to 8% NaCl, while it could tolerate up to 10% NaCl. It showed growth up to 45°C of temperature. The 16S rRNA of isolate OS-1 was submitted to the NCBI Genbank database and accession no. OP881485 was assigned. The phylogenetic analysis showed that strain OS-1 was closely matched to other *P. parafulva* and *Pseudomonas* species (Supplementary Figure 1G). OS-1 showed the swimming, swarming, and twitching motility (Supplementary Figure 1H).

### Genome analysis

A total of 9,516,089 sequencing reads were generated for the strain OS-1. Later assembly correction was done using SPAdes Version 3.15.4 (Nurk et al., 2013) incorporating the results from the Sickle tool, and the scaffold from ABySS to get a draft *de novo* file of about 9.66 MB. Finally the assembled file was subjected to reference (NZ_CP031641) based assembly using CONTIGuator 2 to get the final assembly file of size 5.45 MB. The G + C content of 62% and a total of 71 tRNA were annotated in the genome. Further, gene/protein prediction from the draft genome using the Prokkav1.14 tool (Seemann, 2014) identified a total of 5,127 protein-coding genes.

The genome sequence of OS-1 was further annotated by RAST, and subsystem coverage and non-subsystem coverage generated are 52 and 48%, respectively (Supplementary Figure 2A). RAST analysis predicted the subsystem category distribution (Supplementary Figure 2B) and subsystem feature counts (Supplementary Figure 2C). The top three subsystem features of OS-1 are amino acids derivatives (796 genes), followed by cofactors/vitamins (471 genes) and carbohydrates (319 genes). The other subsystem categories of stress responses (259 genes), protein metabolism (222 genes), fatty acids and lipid metabolism (196 genes), membrane transporter (194 genes), and nucleotide metabolism (157 genes) were observed. The various genes related to sulfur metabolism (Table 2), phosphorous metabolism (Table 3), and metabolism of aromatic compounds (Table 4) were identified.

**Table 2:** Annotated genes for sulfur metabolism in *P. parafulva* OS-1.

Gene name	Functional role
ahpF/C/D	Alkyl hydroperoxide reductase protein
astR	Sulfate ester binding protein
atsK	Putative alkylsulfatase
bcp	Thiol peroxidase
dsrA/B/C	Dissimilatory sulfite reductase
dsrE/F/H	tRNA 5-methylaminomethyl-2-thiouridine synthase
dsrM/K/J/O/P	Sulfite reduction-associated complex
dsrN	Cobyrinic acid A,C-diamide synthase
dsrS	Sulfur oxidation related protein
ssuA	Alkanesulfonates-binding protein
ssuB	Alkanesulfonates ABC transporter ATP-binding protein
ssuD	Alkanesulfonate monooxygenase
tauA	Taurine-binding periplasmic protein
tauA2	Taurine transporter substrate-binding protein
tauB	Taurine transport ATP-binding protein
tauC	Taurine transport system permease protein
tauD	Alpha-ketoglutarate-dependent taurine dioxygenase
trxR	Thioredoxin reductase

**Table 3:** Annotated genes for phosphorous metabolism in *P. parafulva* OS-1.

Gene name	Functional role
corC	Magnesium and cobalt efflux protein
oprO/P	Pyrophosphate-specific outer membrane porin
phnA	Alkylphosphonate utilization operon protein
phnF	Transcriptional regulator
phnK/L	Phosphonates transport ATP-binding protein
phnM	Metal-dependent hydrolase involved in phosphonate metabolism
phoB	Phosphate regulon transcriptional regulatory protein
phoHv	Phosphate starvation-inducible protein
phoP	Alkaline phosphatase synthesis transcriptional regulatory protein
phoQ	Response regulator in two-component regulatory system
phoR	Phosphate regulon sensor protein
phoU	Phosphate transport system regulatory protein
ppK	Polyphosphate kinase
ppX	Exopolyphosphatase
pstA/C	Phosphate transport system permease protein
pstB	Phosphate transport ATP-binding protein
pstS	Phosphate ABC transporter, periplasmic phosphate-binding protein
yggT	Integral membrane protein

**Table 4:** Annotated genes for the metabolism of aromatic compounds in *P. parafulva* OS-1.

Gene name	Functional role
areC	Benzaldehyde dehydrogenase II
areR	Regulator protein
bphA1	Biphenyl dioxygenase alpha subunit
bphB	2,3-dihydroxy-4-phenylhexa-4,6-diene dehydrogenase
bphF	2-hydroxy-3-carboxyhexa-4,6-diene hydrolase
catD	3-Oxoadipate enol-lactonase
catF	Beta-ketoadipyl CoA thiolase
catI/J	3-oxoadipate CoA-transferase subunit
fadA	3-ketoacyl-CoA thiolase
fadB	Enoyl-CoA hydratase
fadD	Long-chain-fatty-acid--CoA ligase
hmgR	Transcriptional regulator
quiA	Quinate/shikimate dehydrogenase [Pyrroloquinoline-quinone]
quiB/C	3-dehydroquinate dehydratase
salA	Salicylate hydroxylase
salD	Putative facilitator of salicylate uptake
salE	Salicylate esterase

### Gene ontology

**Figure 1:**
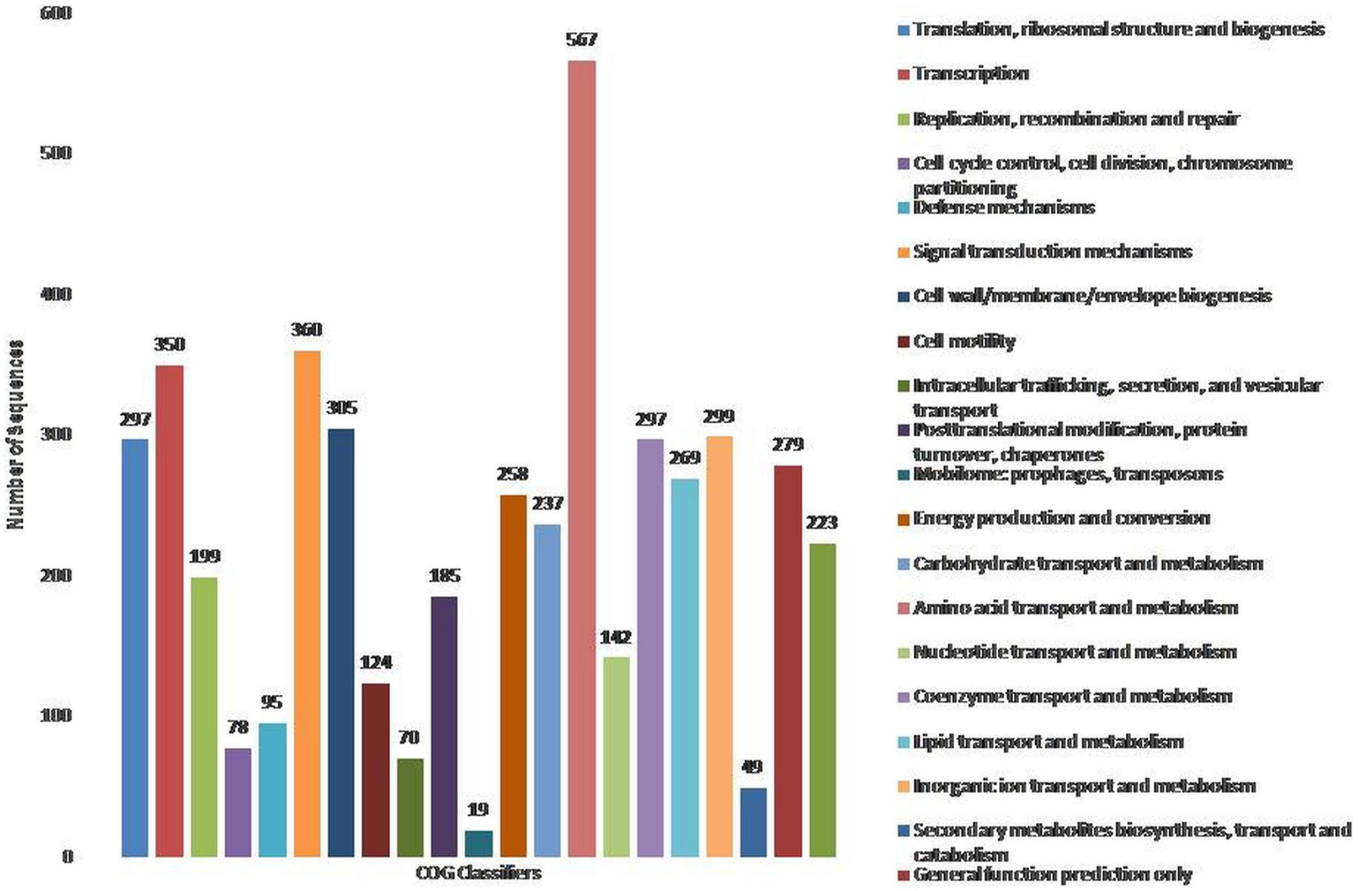
Classification of the cluster of orthologus (COG) functional annotation of *P. parafulva* OS-1 genome. Colored bars indicate a number of genes assigned to each COG functional category.

To assign the functionality of the classified proteins, gene ontology was investigated. A total of 40% of proteins were related to molecular functions, whereas 23 and 37% were observed for cellular components and biological processes, respectively (Supplementary Figure S3). In the molecular functions, 5.19 and 3.90% of proteins were observed for NADH dehydrogenase activity and ubiquinone activity (Supplementary File S1). In the cellular component, 30.23% of proteins were related to the cytosol, followed by the plasma membrane (11.63%) and membrane respiratory chain complex (9.30%) (Supplementary File S1). In the biological processes, 2.82% were related to DNA recombination, as well as cellular and aerobic respiration (Supplementary File S1). The COG database was used for the functional classification of predicted genes, whose distribution within the COG categories is provided in Figure 1. A total of 21 functional annotations was recorded, with the highest number of genes (567) associated with amino acid transport and metabolism, followed by signal transduction mechanism (360), and transcription (350). Similarly, 305 genes were associated with cell-wall/membrane biogenesis, 299 with ion-transporters, and 297 with translation and coenzyme transport, respectively. KEGG analysis identified the genes belonging to the various metabolic pathways (Supplementary Figure S4). The highest number 562 was recorded for different metabolic pathways, 240 with the biosynthesis of secondary metabolites, 132 with microbial metabolisms, 120 for biosynthesis cofactors, 107 with the two-component system, and 96 to ABC transporters. Similarly, 91 were associated with the biosynthesis of amino acids, 69 with carbon metabolism, 47 with purine metabolism, and 42 with flagellar assembly.

### Average nucleotide identity

**Figure 2:**
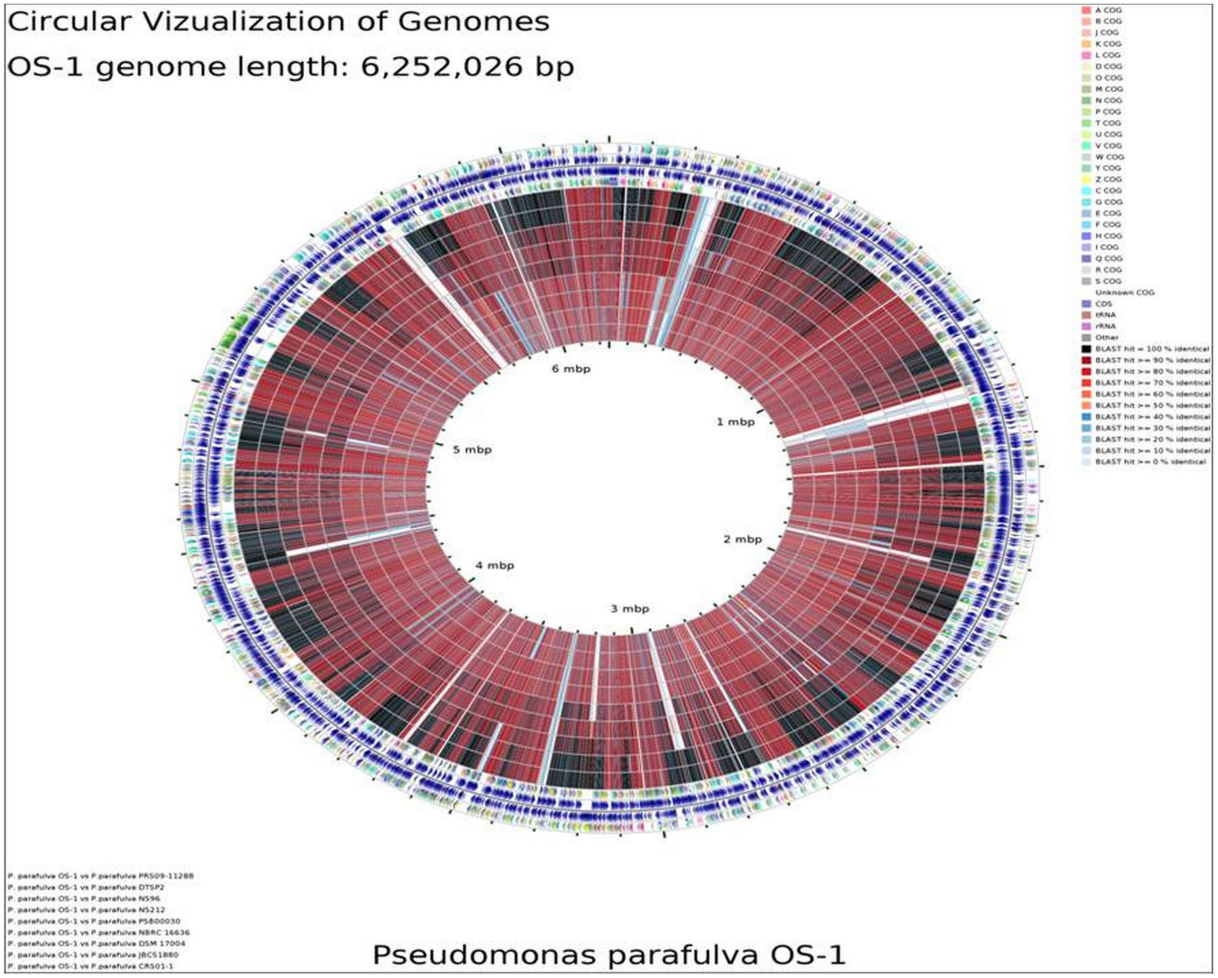
Circular genome map of *P. parafulva* OS-1 compared to other type strains. The percentage similarity is represented by different color codes. The information is read from outer circle to inner as follows: genome size, genes on the forward strand, genes on the reverse strand, t-RNA, r-RNA, GC content, GC skew + and - COG names: Information storage and processing [J] Translation, ribosomal structure and biogenesis [A] RNA processing and modification [K] Transcription [L] Replication, recombination and repair [B] Chromatin structure and dynamics, cellular processes and signaling [D] Cell cycle control, cell division, chromosome partitioning [Y] Nuclear structure [V] Defense mechanisms [T] Signal transduction mechanisms [M] Cell wall/membrane/envelope biogenesis [N] Cell motility [Z] Cytoskeleton [W] Extracellular structures [U] Intracellular trafficking, secretion, and vesicular transport [O] Post-translational modification, protein turnover, chaperones metabolism [C] Energy production and conversion [G] Carbohydrate transport and metabolism [E] Amino acid transport and metabolism [F] Nucleotide transport and metabolism [H] Coenzyme transport and metabolism [I] Lipid transport and metabolism [P] Inorganic ion transport and metabolism [Q] Secondary metabolites biosynthesis, transport and catabolism poorly characterized [R] General function prediction only [S] Function unknown.

**Figure 3:**
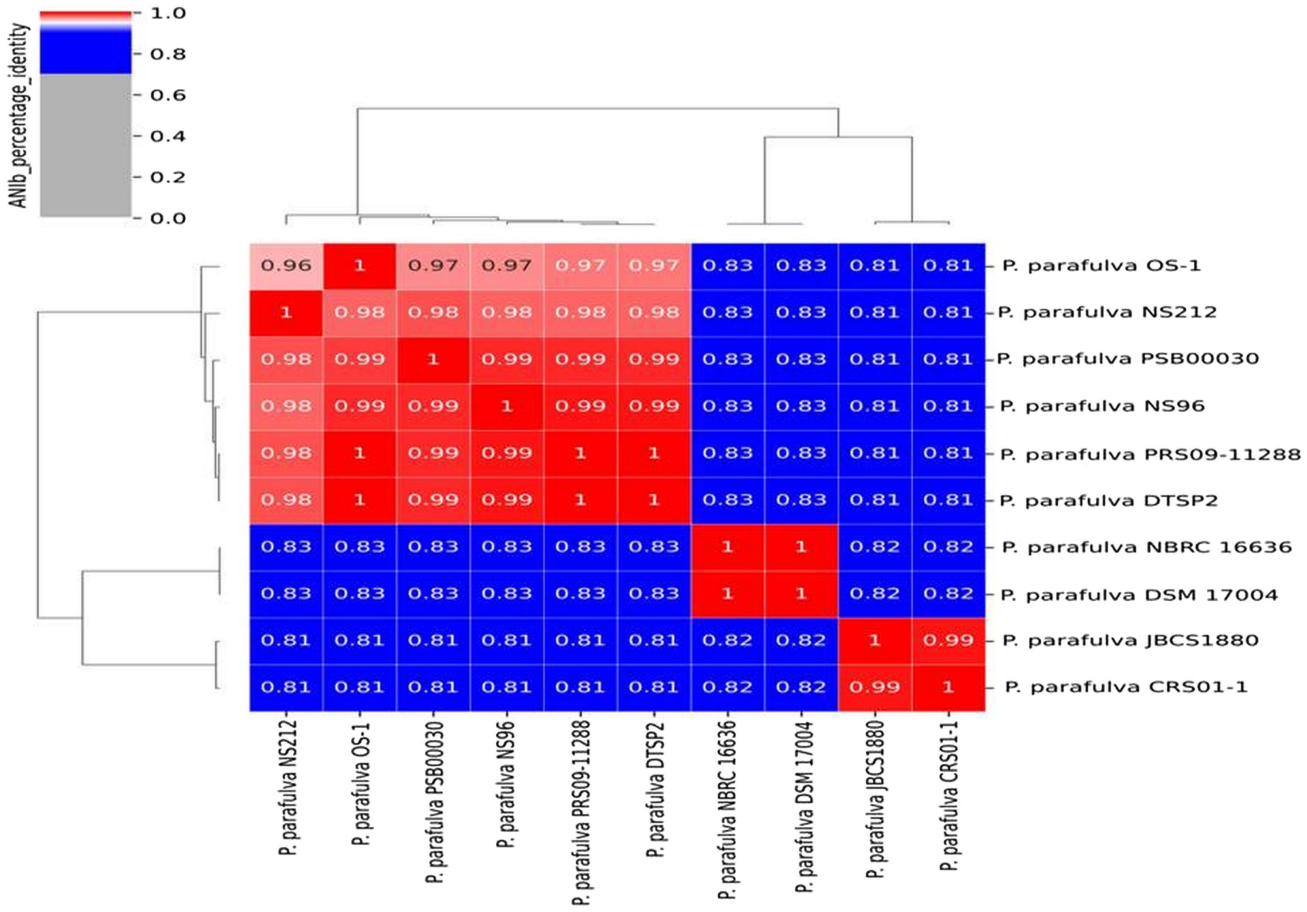
Average nucleotide identity (ANI) matrix showing genomic relatedness performing multi-genome comparison among *P. parafulva* strains from NCBI Database.

BLAST-based alignment of complete genomes of all *P. parafulva* strains was performed to identify the strain closest to the OS-1 isolate. The genome of *P. parafulva* PRS09-11288 and *P. parafulva* DTSP2 was found to be most similar to that of the OS-1 strain, where BLAST percentage identity ranging from 70 to 100% (Figure 2). This similarity is consistent with the results of the whole genome average nucleotide identity (ANI) matrix (Figure 3), where the OS-1 genome shows >96% ANI identity with *P. parafulva* PRS09-11288 and *P. parafulva* DTSP2, clearly suggesting that OS-1 belongs to *P. parafulva* (Figure 3). The detailed information of the genomes used for ANI analysis has been provided in Table 1.

### Patho-genomics analysis

CARD analysis identified the different classes of AMRs which have been summarized in Supplementary Figure 5A. Gene families like resistance-nodulation-cell division (RND) antibiotic efflux pump, major facilitator superfamily (MFS) antibiotic efflux pump, ATP-binding cassette (ABC) antibiotic efflux pump, glycopeptide resistance gene cluster, phosphoethanolamine transferase, small multidrug resistance (SMR) antibiotic efflux pump, multidrug and toxic compound extrusion (MATE) transporter were identified. Additionally, genes associated with NmcA, MSI, YRC and DHT2 beta-lactamase, sulfonamide resistance, chloramphenicol acetyltransferase (CAT), trimethoprim-resistant dihydrofolate reductase (dhfr), streptothricin acetyltransferase (SAT), daptomycin resistance, and fluoroquinolone resistance were observed (Supplementary File S2).

The blast output was filtered based on criteria of minimum 80% identity and subject protein coverage to retain high confidence results where a total of 23 virulence proteins were found having different functions such as endotoxin, motility-associated, antiphagocytosis, and type VI secretion system (Supplementary Figure 5B). In the endotoxin category, genes like waaF (ADP-heptose--lipooligosaccharide heptosyltransferase II), waaG (UDP-glucose: alpha1,3-glucosyltransferase), and waaP (Lipopolysaccharide core heptose I kinase) were observed. Similarly, adherence/motility-associated genes like flgI (Flagellar P-ring protein), fliG (Flagellar motor switch protein), fliM (Flagellar motor switch protein), fleN (Flagellar synthesis regulator), fliP (Flagellar biosynthesis protein), and pilH (twitching motility protein) were observed. Additionally, the T6SS-associated component gene TssC was also observed (Supplementary File S3).

### Biosynthetic gene clusters analysis

The various BGCs in the strain OS-1 genome were annotated using the antiSMASH database. In terms of biosynthetic paradigms, BGCs were comprised of non-ribosomal polypeptides (NRPS) (region 1,185,553 to 1,229,410), O-antigen containing thiopeptide (region 1,531,486 to 1,557,797), siderophore like aerobactin (region 2,971,238 to 2,985,666), and aryl polyenes (region 4,287,759 to 4,331,352) (Table 5).

**Table 5:** The identified BGCs genes in the *P. parafulva* OS-1.

Cluster	Type	From	To	Most similar known cluster	Similarity
1	NAGGN	73,068	87,564	-	-
2	Redox-cofactor	1,861,084	1,882,428	NRP + Polyketide	13%
3	Redox-cofactor	2,708,507	2,730,666	NRP + Polyketide	13%
4	Arylpolyene	3,449,953	3,489,276	Other	40%
5	Arylpolyene	3,517,630	3,558,856	Other	15%
6	NAGGN	5,218,817	5,233,623	-	-
7	Terpene	5,907,470	5,931,109	Terpene	85%

### Comparative genome analysis

A protein coding gene comparison was performed between *P. parafulva* OS-1 and the other genomes of *P. parafulva*. The Venn diagram and the bar plot (Supplementary Figure S6) showed that the numbers of core ortholog clusters shared by all the six species were 5, that suggests their conservation in the lineage after speciation events. The cumulative number of ortholog clusters shared between any two genomes, including the OS-1 was 1,998. A total of 32 gene clusters were unique to only a single genome. These clusters are probably gene clusters within multiple genes or in-paralog clusters which suggest that a lineage-specific gene expansion has occurred in these gene families. The cumulative number of ortholog clusters shared between any two genomes, including the OS-1 was 1740 (Supplementary Figure 6A). Additionally, the bar plot below the Venn diagram showed that the number of ortholog clusters for each species varied; *P. parafulva* OS-1 (2,935), *P. parafulva* PRS09-11288 (3,168), *P. parafulva* strain DTSP2 (5,23), *P. parafulva* strain NS212 (4,84), *P. parafulva* strain PSB00030 (2,87), and *P. parafulva* strain NS96 (2,54) (Supplementary Figure 6B). The pairwise heatmap was performed for *P. parafulva* OS-1 and other strains to highlight the overlapping number of protein clusters (Supplementary Figure 6C). A red color gradient showing the highest overlapping protein cluster thresholds was noted between *P. parafulva* OS-1 and *P. parafulva* 11,288. Island viewer identified 22 genomic islands in the OS-1 genome. Interestingly, there were 16 ORFs belonging to the virulence factors and antibiotic resistance (Supplementary Figure S7).

### CAZymes analysis

**Figure 4:**
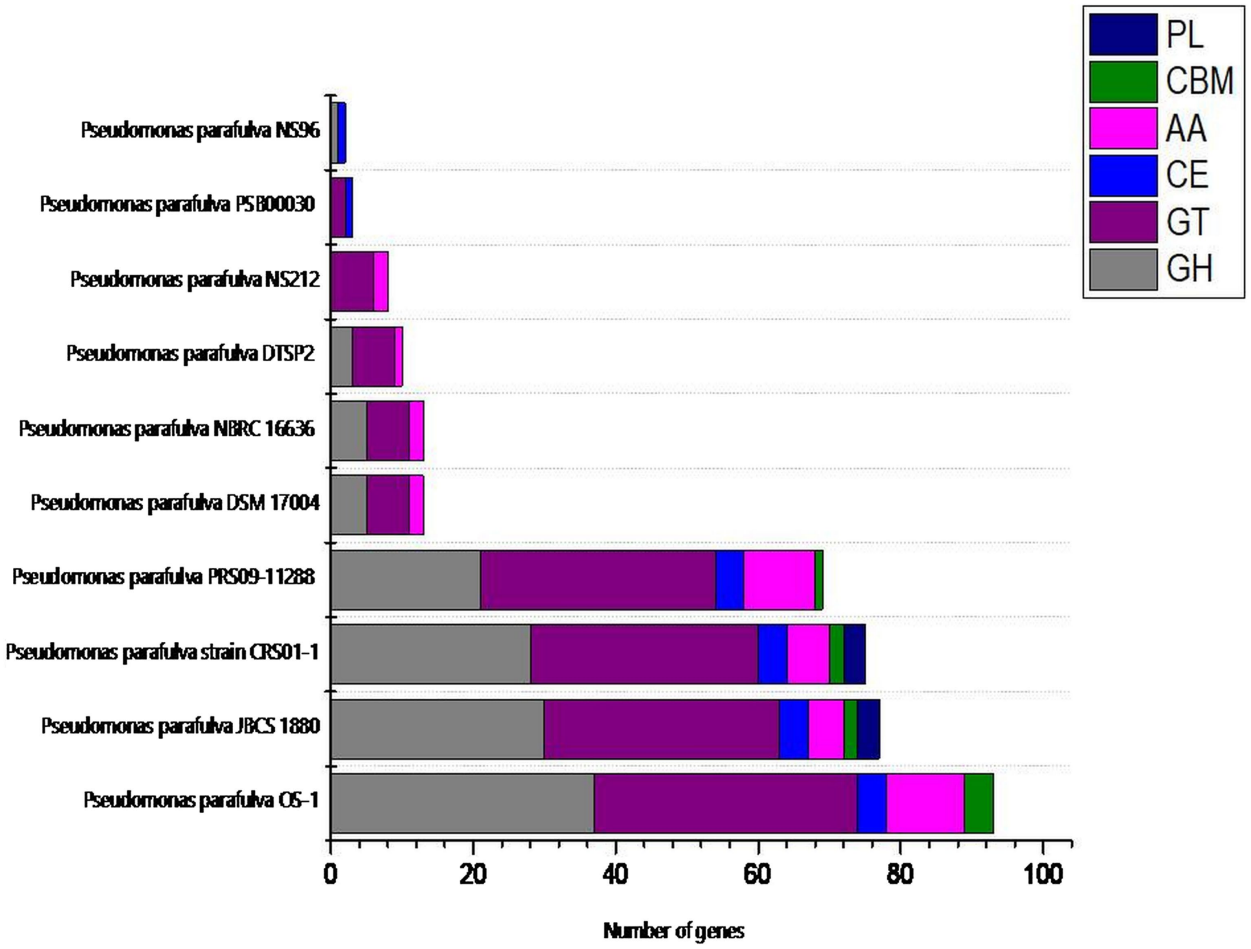
The distribution of various CAZymes like carbohydrate-binding modules (CBMs), glycoside hydrolases (GHs), glycosyl transferases (GTs), polysaccharide lyases (PLs), carbohydrate esterases (CEs), and auxiliary activities (AAs) in *P. parafulva* OS-1, and its comparison to other *P. parafulva* strains.

To investigate the industrial-relevant enzymes involved in the breakdown of complex carbohydrates, the OS-1 genome was analyzed by the dbCAN2 server. As a result, 92 CAZymes genes were identified in the OS-1 genome, which was classified into glycoside hydrolases (GHs), glycosyltransferases (GTs), carbohydrate-binding molecules (CBMs), carbohydrate esterases (CEs), and auxiliary activities (AAs). Of these, the most abundant CAZymes were GHs and GTs with an equal 37 genes, followed by AAs (10 genes), CBMs (4 genes), and CE (4 genes) (Figure 4). The different groups of CAZymes were also compared to other *P. parafulva* genomes (Supplementary Table S3), and a comparison of different CAZymes has been demonstrated in Figure 4. A very low level of CAZymes was observed for *P. parafulva* NS96 and *P. parafulva* PSB00030.

### Pan-genome analysis

**Figure 5:**
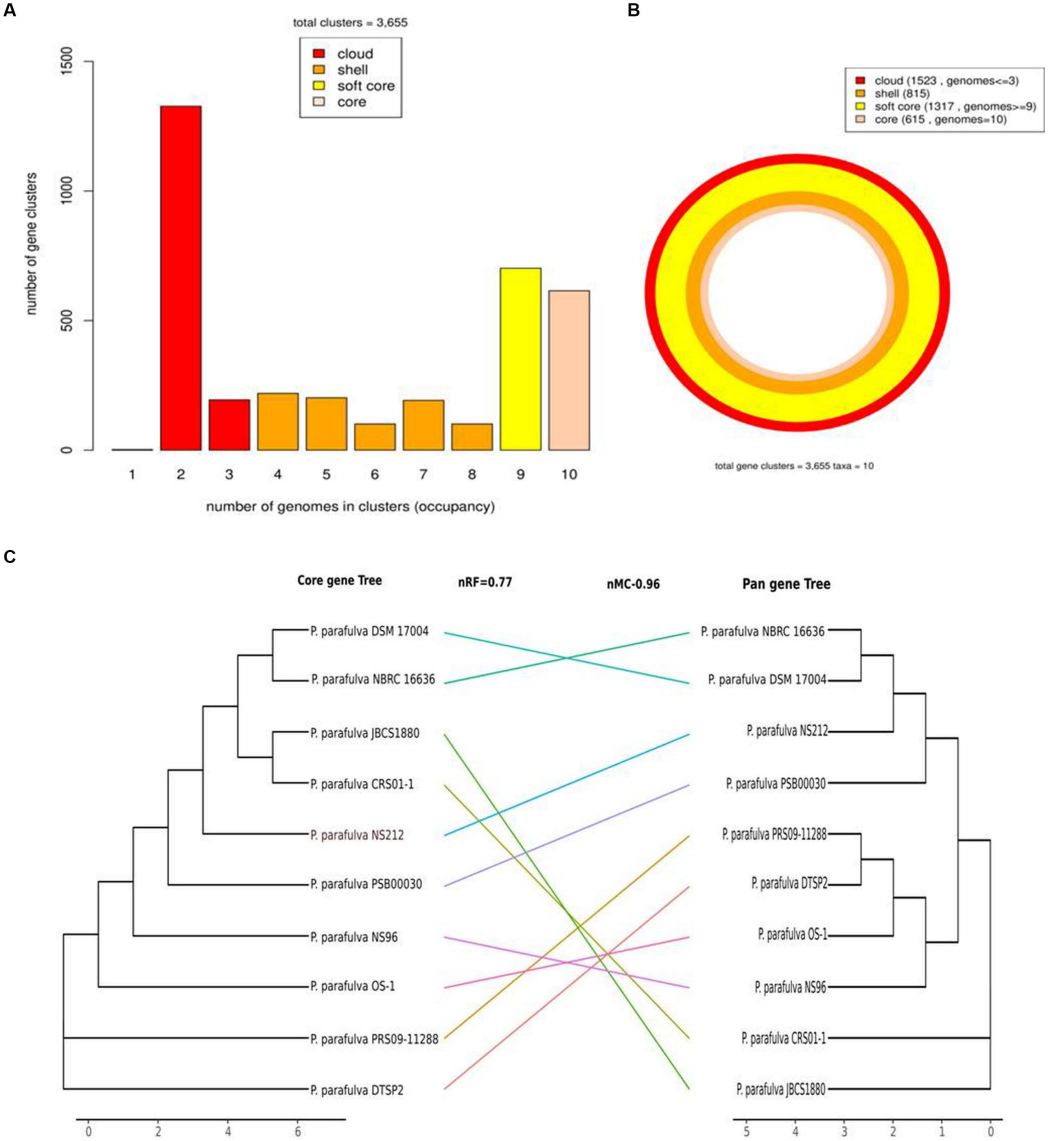
Graph of the pan-genome distribution of *P. parafulva* genomes; **(A)** Bar plot of pangenome distribution; **(B)** Circular plot of pan genome: innermost circle (in light pink) represents core genome. The 2nd innermost circle (in dark orange) represents the gene in the shell, whereas 2nd outermost circle (in yellow) represents the soft core and the outermost circle (in red) represents cloud genes that are unique or present in a maximum of two genomes; **(C)** Comparison between the core-genome and pan-genome tree. Normalized Robinson-Foulds (nRF) and normalized matching cluster (nMC) scores were used to measure the congruence of the two trees.

All publicly available *P. parafulva* genome sequences were retrieved from the NCBI database. The size of all *P. parafulva* genomes including our strains was ranging from 4.6 Mb to 5.4 Mb and GC contents from 61.71 to 63.46 percent (Table 1). A total of 3,655 pan-gene families were observed among *P. parafulva* genomes. The *P. parafulva* genome components were defined as core genome (genes present in all genomes), soft-core genome (genes present in at least 95% of the genomes), cloud genome (genes present only in one or three genomes), and shell genome (remaining moderately conserved genes present in several genomes) (Figure 5A). In this *P. parafulva* dataset, the core-genome is composed of 615 gene clusters, soft-core by 1,317, the shell genome by 815, and the cloud genome by 1,523 (Figure 5B). Shell and cloud genes constitute accessory genetic content that attributes to genetic diversity and adaptive capability of isolates such as environmental adaptation, drug resistance, and host adaptation (Medini et al., 2005). In *P. parafulva*, the accessory genetic content holds 2,338 gene clusters, of which 1,523 gene clusters are present only in one or three genomes. This observation indicates despite being analyzed with a very low number of genomes, *P. parafulva* have reduced core-genome components compared to their variable counterparts. The functional annotation demonstrates *P. parafulva* OS1 genome contains a number of genetic determinants that confers several advantages to the isolate, which include environmental adaptation (osmotic stress adaption, stress protection and cold and heat shock), drug resistance, and host adaptation (anti-microbial resistance, virulence or biofilm-forming proteins), and metal resistant phenotype. The diverse metabolite biosynthesis capability of *P. parafulva* OS1 isolate and increasing pan-genomic content of *P. parafulva* genomes corroborate with the previous pan-genome-based finding on wider metabolite machinery and pathogenicity of *Pseudomonas* group (Freschi et al., 2019; Alba et al., 2021).

**Figure 6:**
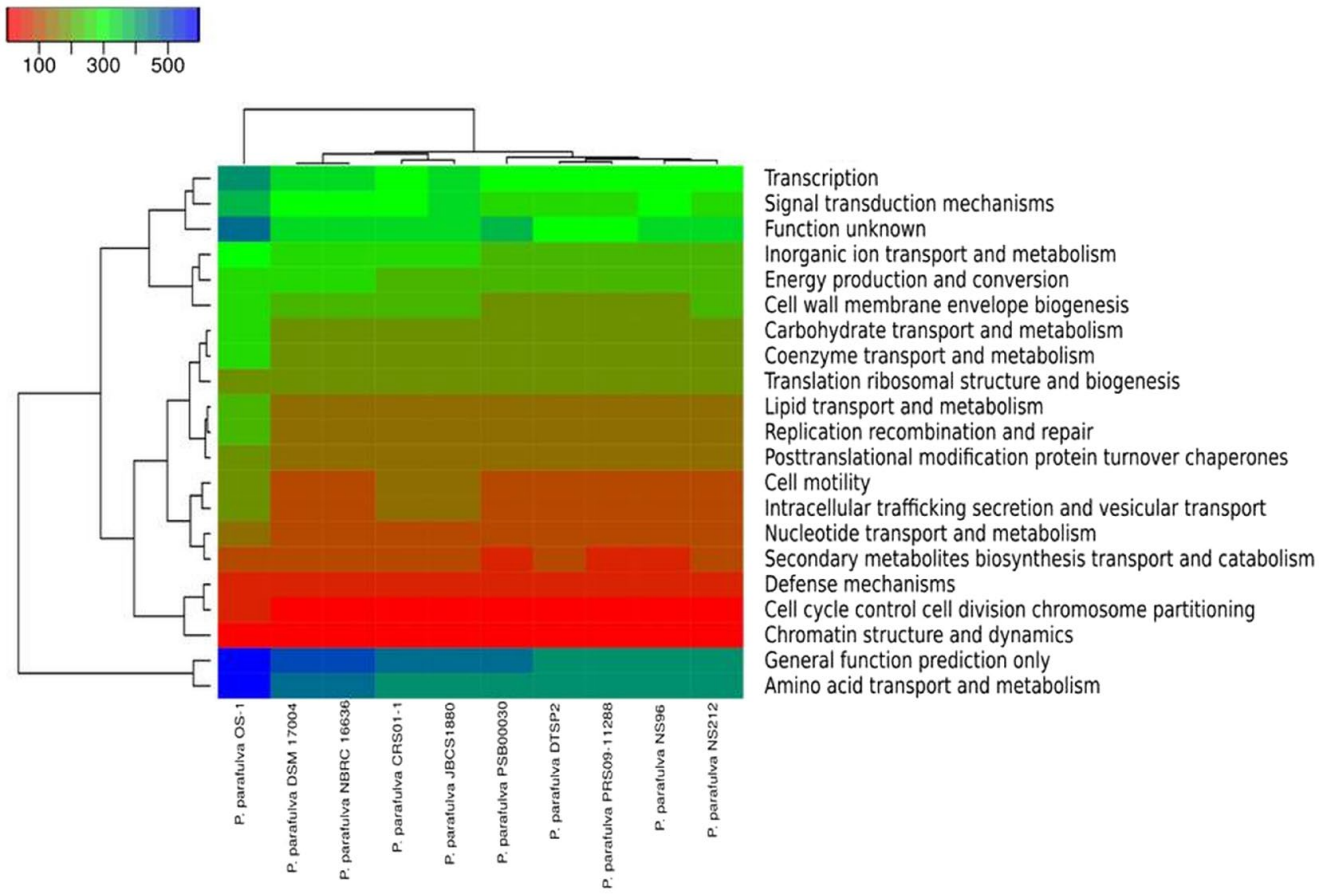
The heatmap (COG functional) represents the distribution of the functional abundance of differentially enriched metabolic functions in *P. parafulva* genomes. Heatmap showing the normalized relative abundance of the clusters of orthologous groups (COG) categories enriched in the protein-coding genes in the selected *P. parafulva* genomes. The strains and COG categories were clustered using the Manhattan distance. The color scale represents the relative abundance of gene content for each category, normalized by the sample mean.

The phylogeny of the core and pan-genome was constructed to draw inferences on the genetic evolution of the core as well as variable genes of 10 *P. parafulva* genomes. Comparison between core and pan-genome trees highlights different branching order and clustering patterns of *P. parafulva* strains (Figure 5C). This distinct evolutionary signal in core and pan-genes of *P. parafulva* genomes was assessed using nMC and nRF score (Robinson and Foulds, 1981; Bogdanowicz et al., 2012). These scores range from 0 to 1 and if the value is close to 1 that indicates higher dissimilarity in branching order between two evolutionary trees. Clustering of *P. parafulva* strains based on their diverse metabolic functions reveals the OS-1 strain as a separate individual with a distinct metabolic profile from the rest of the *P. parafulva* strains (Figure 6). A close look into functional abundance demonstrated amino acid metabolism and general function are the most abundant functions in *P. parafulva* strains followed by transcription, signal transduction, ion transport, energy production, and cell wall biogenesis (Figure 6).

## Discussion

Previous studies have characterized the *P. parafulva* PRS09-11288, *P. parafulva* JBCS1880, and *P. parafulva* DSM 17004 T isolates from rice fields (Uchino et al., 2001), whereas, *P. parafulva* YAB-1 was isolated from perfluorinated compound-contaminated soils (Yi et al., 2015). In this study, test isolate OS-1 was isolated from the waste-contaminated soil sample and identified as *P. parafulva* following 16S rRNA sequencing. The phenotypic characterization and antibiotic susceptibility patterns are insufficient to provide insight into the various genes repository for different characteristics. Therefore, we followed the genomic approach to uncover detailed gene analysis. The genome analysis of microbes inhabiting different ecological niches provides an opportunity to understand the molecular mechanism involved in survival and is particularly useful in the identification of key genes/genetic traits involved in survival strategies (Ward et al., 2008; Baquero et al., 2021). Furthermore, comparative genome analysis can address the genetic relatedness between phylogenetic similar species inhabiting diverse environments and provide important information on relevant traits between functionally important genetic elements constituting the core genome (Sinha and Krishnan, 2021). The ANI analysis is commonly used for calculating the genomic distance and establishing accurate phylogenetic evolution, which could be helpful to overcome the challenges caused by horizontal gene transfer (HGT) and mutation events (Konstantinidis and Tiedje, 2007). The establishment of an accurate phylogenetic tree supports the understanding of the major transition events in evolution (Emms and Kelly, 2015; Kapli et al., 2020). Additionally, ANI analysis presents a critical parameter for bacterial species delineation (Rosselló-Móra and Amann, 2015).

The ANI analysis of *P. parafulva* genomes shows six out of 10 genomes followed the ANI threshold value (> = 96%), but four genomes (*P. parafulva* NBRC 16636, *P. parafulva* DSM 17004, *P. parafulva* JBCS1880 and *P. parafulva* CRS01-1) are not consistent with ANI and showing lower values compared to rest of the *P. parafulva* genomes (Figure 3 ANI_matrix). However, these four genomes form groups of two ANI clusters having more than 96% of ANI values. This observation implies that there may be a wrong taxonomy assignment of *P. parafulva* genomes and highlights the necessity of taxonomic reclassification of genomes submitted under *P. parafulva* species (Jun et al., 2015). Further to get more clarity, we included one very closely related outlier species *P. fulva* YAB1 into the ANI matrix to decipher if disputed *P. parafulva* species are misnamed as *P. parafulva* while they belong to the member of *Pseudomonas* genus. However, we observed that the disputed *P. parafulva* strains do not belong to *P. fulva* (showing ANI < 96%) group (Supplementary Figure S8), probably suggesting these strains may be associated with another distinct subgroup of the *Pseudomonas* genus. Altogether it can be assumed that the exact information on *Pseudomonas* subpopulation that belongs to disputed *P. parafulva* strains is still unclear and highlight the need for in-depth taxogenomic analysis of *Pseudomonas* genus.

Additionally, we assume that isolate OS-1 might be a strong recipient of HGT event, which leads to expansion of its genome size and also due to less availability of *P. parafulva* genome in a public database, gives opaque information about its genome size or genomic content. Therefore, it leaves the scope for new additional genomic information, especially to *P. parafulva* species. The presence of genomic islands, prophages with large (>9 kb) integrative elements and repeat elements in the OS-1 genome indicates that HGT might happen during evolution (Supplementary Figure S8). HGT through mobile genetic elements (MGEs) such as plasmids and genetic islands increase the virulence and pathogenic repository (Sobecky and Hazen, 2009). Bartoli et al. (2016) suggested that the acquisition of new DNA sequences through HGT finally enhances the pathogenicity in bacteria.

### Cog

COG analysis demonstrated a high proportion of genes belonging to amino acid metabolism categories suggesting that OS-1 strain has a well-developed system for the transport and metabolism of a wide spectrum of carbon and nitrogen sources, which represent an essential feature for industrial producers of bulk chemicals (Szczerba et al., 2020). The presence of a high number of transporter genes is an essential feature for the industrial producers of bulk chemicals. Furthermore, the high percentage of genes involved in the transcription process ensures the high metabolic activity of the OS-1 strain (Szczerba et al., 2020).

### Osmotic stress adaption

Based on RAST analysis with the SEED database, various genes responsible for resistance to osmotic stress were found. Many genes involved in osmoregulated periplasmic glucans like glucans biosynthesis glucosyltransferase (MdoH), glucans biosynthesis protein (MdoG, MdoC, MdoD), phosphoglycerol transferase (MdoB), and cyclic beta-1,2-glucan synthase (NdvB) were identified. The OpuA system is the predominant transporter for glycine betaine and consists of three components: an ATPase (OpuAA), an integral membrane protein (OpuAB), and a hydrophilic polypeptide (OpuAC) (Wargo et al., 2008). The OpuC glycine betaine uptake system is related to OpuA but contains an additional integral inner membrane component. Both OpuA and OpuC exhibit structural and functional similarities to the ProU system from *E. coli* (Wargo et al., 2008). Upon osmotic stress, ProU is also involved in proline and betaine uptake, which both play a role in osmoadaptation in *E. coli*. Annotation of the OS-1 genome identified the various genes involved in the protection of reactive oxygen species. Genes like superoxide dismutase (sodA, sodB) are involved in dismutating O_2_^−^ to molecular oxygen (O_2_) and hydrogen peroxide (H_2_O_2_) at rates nearly sufficient to limit diffusion (Verneuil et al., 2006). The OS-1 genome harbored the genes for glutathione synthetase (GSH; gshA, gshB, gshF, gshFB), which performs diverse functions including free radical scavenging, redox reactions, formation of deoxyribonucleotides, detoxication of xenobiotics, amino acid transport, and many others (Copley and Dhillon, 2002). The presence of major detoxification enzymes like glutathione transferases (GST) protects the bacteria from attack by reactive electrophiles (Kanai et al., 2006). Genes like Lactoylglutathione lyase (GloA) and Hydroxyacylglutathione hydrolase (GloB) catalyze the two sequential reactions of the glutathione-dependent pathway of methylglyoxal detoxification (Korithoski et al., 2007). Genes like Phytochelatins (PCS) are known to be the main heavy-metal-detoxifying peptides in few microorganisms, and hydrolyze GSH and GS-conjugated xenobiotics.

### Metal-resistance genes

Referring to the genome annotation analysis, OS-1 strain showed a good number of metal resistance genes (Supplementary Table S4). One major mechanism underpinning microbial resistance against both heavy metals and antibiotics is by effluxing out these compounds. In the genome of the OS-1, a czc operon comprising three structural genes, czcC, czcB, and czcA was found. The czc operon proteins constitute metal-transporting resistance-nodulation-division-type (RND type) efflux systems, corresponding to the first layer of metal resistance in bacteria (Ducret et al., 2020). The RND family plays a key role in the heavy metal resistance of gram-negative bacteria and function as a tripartite complex composed of the inner membrane, periplasmic, and outer membrane components (Fernando and Kumar, 2013). The CzcCBA efflux system consists of CzcC (outer membrane protein), CzcA (inner plasma membrane transport protein), and CzcB (membrane fusion protein). Overall, the CzcCBA efflux pump functions to transport the divalent cation/proton antiporters, and is also responsible for the detoxification of Zn^2+,^ Co^2+^, and Cd^2+^ (Legatzki et al., 2003). At the genome level, OS-1 possessed an arsenal of efflux pumps about metal resistance, cobalt-zinc-cadmium resistance protein (CzcA), heavy metal RND efflux outer membrane protein (CzcC family), cobalt-zinc-cadmium resistance protein (CzcD), zinc transporter (ZitB), Zn(II) and Co(II) transmembrane diffusion facilitator (CzrB), and copper efflux system protein (CusB).

Furthermore, the OS-1 genome also contained the CopA and CopB P-type Cu^+^-ATPases, which are responsible for transporting monovalent Cu from the cytoplasm (Bondarczuk and Piotrowska-Seget, 2013) and is reported in many gram-negative bacteria, e.g., *Pseudomonas* sp., (González-Guerrero et al., 2010), *Vibrio cholera* (Marrero et al., 2012), and *Salmonella typhimurium* (Osman et al., 2010). In addition, genome analysis of the OS-1 strain revealed the presence of the cueR gene encoding CueR, which regulates the expression of the P-type Cu^+^-ATPase (Marrero et al., 2012). Additionally, the OS-1 genome also possesses the arsenate resistance arsRBC operon which encodes arsR (a transcriptional regulator), arsB (integral membrane protein that extrudes arsenite from the cell cytoplasm), and arsC (an arsenate reductase to transform arsenate to arsenite) (Ben Fekih et al., 2018). Additionally, OS-1 genome carried the arsH gene encoded ArsH protein, conferring resistance to methyl As(III) derivatives in *P. putida* and *S. meliloti* (Chen et al., 2015). It is also worth emphasizing that the OS-1 genome carries the merE gene which encodes a broad mercury transporter that mediates the transport of both Hg^2+^ and CH_3_Hg(I) across bacterial membranes (Kiyono et al., 2009).

### Stress protection

RAST-based functional annotation founds several key elements associated with the adaption of OS-1 to various niches. Strain OS-1 harbored several elements associated with stress protection like alkyl hydroperoxide reductase (AhpC), fumarate and nitrate reduction regulatory protein (FnrR), nitrite-sensitive transcriptional repressor (NsrR), and redox-sensitive transcriptional activator and regulator (SoxR & SoxS) (Roca et al., 2008). Additionally, SAM family enzymes are involved in the heat shock gene cluster, and DnaK family chaperones participate actively in the response to hyperosmotic and heat shock by preventing the aggregation of stress-denatured proteins (Mayer, 2021). Additionally, CspA family cold-shock proteins that are induced in response to temperature downshift and preventing secondary structure formation to facilitate translation at low temperatures were noted (Keto-Timonen et al., 2016).

Genes associated with periplasmic stress responses (DegS, DegQ, RseB, RseP) and periplasmic chaperones such as (Skp, SurA) were identified. These periplasmic genes together with chaperones relieve the periplasmic stress (Sklar et al., 2007; Kim et al., 2021). Gene related to ATP-binding cassette (ABC) transporters like phosphate (*PstABCS*), zinc (*ZnuABC*), cobalt (*Cbi MNQO*), and molybdenum (*ModB*) were detected which indicate that strain is able to resist metal stress and tolerate the heavy metal environments. These ABC-transporter proteins contribute to the high level of resistance against metal by pumping the metal ions out of the cells (Gaba et al., 2020) and were found to be differentially regulated by more than one metal ion (Tame et al., 1994).

### Cold and heat shock

A group of related proteins that are induced in response to temperature downshifts, enable cells to adapt to cold temperatures. Multiple genes (9 genes) coding for different classes of cold shock protein (CspA, CspB, CspC, CspD, CspE, CspF, CspG, CspH, and CspI) which may help the strains cope with temperature variations were identified. These CspA families of cold-shock proteins may function as RNA chaperones to prevent secondary structure formation and facilitate translation at low temperatures (Keto-Timonen et al., 2016). The CspA homologs have also been shown to facilitate transcription anti-termination, important in reprogramming gene expression. Similarly, multiple genes coding for a heat-shock protein (DnaK, DnaJ, GrpE, and MiaB) were identified. DnaK forms chaperone machinery with co-chaperones DnaJ and GrpE, which participates actively in the response to hyperosmotic and heat shock by preventing the aggregation of stress-denatured proteins and by disaggregating proteins (Chae et al., 2004). DnaK operons are currently unknown, it is noteworthy that many of them are predicted to be involved in various tRNA or rRNA modifications. Since many tRNA modifications are believed to improve reading frame maintenance (Urbonavicius et al., 2001), it is tempting to speculate that the role of these additional proteins is protecting ribosomal function (e.g., accuracy of translation) during heat shock and other stressors.

MiaB-like enzymes are specifically over-represented in certain thermophiles, such as *Thermotoga maritima*, *Aquifex aeolicus,* and *Methanococcus jannaschii*, suggesting that they might additionally participate in some thermophile-specific nucleotide modifications (Anantharaman et al., 2001). MiaB is a bifunctional radical-S-adenosylmethionine enzyme that belongs to isopentenyl-adenosine tRNA methylthiolase that assists in the prevention of frameshift mutations (Pierrel et al., 2004). The putative heat-shock-related proteins are potentially involved in improving ribosomal function during stress, which is further supported by the fact that “GTP-binding protein LepA” is often co-localized with “extended dnaK gene clusters.” This universally conserved protein has been recently shown to function as an additional elongation factor required for back-translocating posttranslational ribosomes. LepA recognizes ribosomes after a defective translocation reaction and induces a back-translocation, giving EF-G a second chance to translocate the tRNAs correctly (Qin et al., 2006).

### Bacterial growth

The metabolic pathways genes for carbohydrates, lipids, amino acids and nucleotides, and cofactors and vitamins were identified in the OS-1 genome which supports bacterial growth in nutrient limitation or even under adverse conditions. In the OS-1 genome, we identified the genes involved in the metabolism of acyl sugar, monocyclic and acyclic monoterpenes, and carbohydrate esterase family I. Acyl sugars with long-chain esters are involved in the insecticidal activity (Fan et al., 2019), whereas carbohydrate esterase family-I is involved in the removal of esters from the carbohydrate core (Nakamura et al., 2017). Terpenoids are associated with antimicrobial activity and bacteria can metabolize them for further use as carbon sources (Huchelmann et al., 2017; Schuurink and Tissier, 2020). The ability of nitrogen metabolism was evidenced by the presence of genes involved in assimilatory and dissimilatory nitrate reduction (ANR and DNR) (Huang et al., 2020), glutamate metabolism (Paul et al., 2007) and cyanide metabolism, respectively (Lin et al., 2017).

The OS-1 genome also showed the presence of pyoverdines type siderophore to access assimilable iron (Fe^+2^). The production of pyoverdines is associated with pathogenicity in *P. aeruginosa* and *P. syringae* (Taguchi et al., 2010; Kang et al., 2018). It is also related to the promotion of plant growth and disease control in *many Pseudomonas* spp. that were associated with the plants (Aznar and Dellagi, 2015; Berendsen et al., 2015; Trapet et al., 2016). Another interesting genomic trait of OS-1 is the presence of several gene clusters associated with the metabolism of aromatic compounds. The presence of metabolic pathway genes such as HPD hydratase (bphE1), 4-hydroxy-2-oxovalerate aldolase (bphF1), and acetaldehyde dehydrogenase (acylating) (bphG) metabolize biphenyl through the 2-hydroxypenta-2,4-dienoate (HPD) and benzoate metabolic pathways, suggests its diverse metabolic potential (Sakai et al., 2003). The presence of alkane sulfonates is used in sulfur utilization and atsK gene of *Pseudomonas* spp. is required for growth with alkyl sulfate esters as sulfur source (Eichhorn et al., 2000). The secreted phosphodiesterases and phosphomonoesterases including PhoD, PhoB, and PhoA, are believed to have a role in the teichoic acid degradation, thereby, providing an additional phosphate supply for uptake via the PstS high-affinity transport system (Paul et al., 2004).

### Metabolism

In the OS-1 genome, various genes related to the phosphate transport system regulator (Pho regulon) were identified. Genome annotation also identified a high-affinity Pi transport system (PstS system) and a family of alkaline phosphatases (PhoA, PhoB, PhoD, and PhoP), whose function is to regulate the decreasing Pi pool. Under phosphate-limiting conditions, phosphorylated PhoP (PhoP~P) activates the tua genes, thereby initiating the synthesis of a non-phosphate-containing polymer like teichuronic acid (Archibald et al., 1993). The secreted phosphodiesterases and phosphomonoesterases like PhoA, PhoB and PhoD play an important role in teichoic acid degradation, thereby, providing the phosphate supply for uptake via the PstS high-affinity transport system (Paul et al., 2004). The presence of polyphosphate kinase (PPK) catalyzes the reversible transfer of the terminal phosphate of ATP to form a long-chain polyphosphate (polyP) which is a ubiquitous molecule formed by phosphate (Pi) residues linked by high-energy phosphoanhydride bond (Paul et al., 2004). Genes involved in ABC-phosphonate transporter (phnCDE, psiD) were identified which are involved in alkyl phosphonate uptake (Martín and Liras, 2021).

In OS-1 genome, dsr gene clusters (DsrA, DsrB, DsrC, DsrE, DsrF, DsrH, and DsrR) were recorded which are particular functions in sulfate and sulfide oxidation in many microorganisms. A previous study showed the role of DsrR in the regulation of sulfur oxidation in *Allochromatium vinosum* (Grimm et al., 2010). Selenium, a naturally occurring element, is essential for biological systems at low concentrations but toxic at higher levels. High concentrations of selenium oxyanions are highly toxic and mutagenic (Kessi et al., 2022). Many organisms, therefore take up selenate and/or selenite from their environment, and detoxify them by reduction and/or methylation of selenate and selenite. In the genome of OS-1, genes related to selenium uptake (CysA, DedA) and transport (YbaT, NmpC) were noted. Formaldehyde is produced at significant levels by abiotic and biological processes, and is an intermediate metabolite formed during the metabolism of methanol or other methylated compounds (Wilson et al., 2008). Because this compound can inactivate many cellular components, the OS-1 genome harbored the GSH-dependent pathway genes (FGH, FrmR, Reg-F) to metabolize/detoxify formaldehyde (Barber and Donohue, 1998).

### Gene-related to biotechnological applications

The RAST analysis identified various genes related to industrial applications and potential in biotechnology including phosphates, sulfatase, phosphoesterase, and proteases. These secreted extracellular enzymes favored the microbes in survival under harsh conditions like high temperature, salinity, osmotic stress and the presence of toxic compounds (Kamalanathan et al., 2018). Additionally, these enzymes are involved in nutrient cycles of carbon, phosphorus, nitrogen and sulfur molecules, and also degrade the complex molecules (Chung et al., 2020). The secreted exopolysaccharides (EPS) are involved in the detoxification of heavy metals, removal of industrial-relevant pollutants, and act as anticoagulants and immunomodulators (Shukla et al., 2017). The strain also harbored biosynthetic genes related to valuable compounds like squalene which play diverse roles as an antioxidant, anticancer and antibacterial agent, health care products, and adjuvant for vaccines (Gohil et al., 2019). CAZymes are involved in the breakdown of complex carbohydrates and glycoconjugates during biological processes (Gavande et al., 2021). Therefore, genes encoding various CAZymes were investigated and identified several genes involved in the breakdown of complex glycans.

### Colonization

OS-1 bacterial colonization begins with the attachment of planktonic cells to the surfaces followed by adhesion, micro-colonies development, and extracellular matrix production. The developed biofilm contains high cell density with a high metabolic capacity to develop new microcolonies (Grinberg et al., 2019; Chaudhry et al., 2021). The aforementioned was consistent with identified genes involved in antimicrobial metabolites production, and bacterial chemotaxis in *P. parafulva* OS-1. Chemotaxis is a fundamental process that requires organized chemosensors and signal transduction mechanism to activate and control the flagellum movement which favors bacterial survival in an unfavorable environment (He and Bauer, 2014; Sampedro et al., 2015; Scharf et al., 2016). *P. parafulva* OS-1 genome annotation revealed many genes participate in the chemotaxis mediated by flagella, pilli, and genes involved in biofilm formation.

### Pan-genome analysis

ANI is a nucleotide-level measure of genomic similarity between the coding regions of two genomes, it only calculates the percentage of residue-to-residue level similarity solely used for taxonomic demarcation. On the contrary core and pan genomes represent consistent and variable gene repertoire among the individuals of the same species mainly used to decipher gene content evolution and adaptation of organisms. The pan-genome analysis of OS-1 was conducted to explore the physiological as well as metabolic diversities and further expound upon the lifestyle attributes (Chun et al., 2019). The core genome contributes to the basic physiology of the organism and thereby represents a set of genes common to all species (Rouli et al., 2015). The accessory genes represent the strain-specific unique genes and are not found in more than one organism (Chaudhari et al., 2016). The higher number of cloud genes indicates increased genetic heterogeneity among the *P. parafulva* strains suggesting the open pan-genomic nature of *P. parafulva* species (Figure 5). The growing repertoire of the variable genetic content of *P. parafulva* species makes them competent to survive in various ecological habitats and indicates the possibility of widening their range of habitats. Additionally, the lack of congruence between the pan and core gene phylogenetic tree implies the expansion of variable genetic content in *P. parafulva* group through various means of gene acquisition from its host or outside environment. It is evident from the analysis that *P. parafulva* OS-1 genome is functionally more enriched than any of the *parafulva* strains reported to date (Figure 6). We performed Get-homolog embedded Pfam annotations of core, soft core, shell and cloud genes to get the insight of functional profile of different pan-genome categories (Supplementary File S4). Pfam annotation shows the following function in each pan genome category, Soft core: transporter genes, permease genes, regulator genes and ribosomal proteins; Shell: type IV secretion system, chaperons and hypothetical proteins; Cloud: transporter, antibiotic efflux, response regulator, metal-binding protein and hypothetical proteins; Core: beside housekeeping genes it includes genes of heavy metal sensor, heavy metal response, various exporter and importer genes, and carbohydrate utilization genes. In summary, it is evident from the analysis that each pan-genome category shares overlapping gene functions, the cloud gene category is relatively functionally more enriched which is consistent with its number of genes. The presence of chaperons, transporter, permease in pan-genomes, and various heavy metal sensor and response genes in both core and pan genome sections indicates the probable capability of strong habitat adaptation of *P. parafulva* species. In comparison to other strains, OS-1 encodes a few functions with higher proportions such as transcription, carbohydrate transport and metabolism, coenzyme transport and metabolism, lipid transport and metabolism, replication recombination and repair, and amino acid transport and metabolism (Figure 6).

MLST-based analysis has demonstrated that *P. parafulva* mainly originated from *P. putida* phylogenetic group, and is very closely associated with *P. fulva* and *P. cremoricolorata* type species (Mulet et al., 2012). Variation in recombination rates usually creates difficulties for species clustering based on individual genes or 16S rRNA, this can be addressed by the multilocus sequence typing (MLST) approach (Maiden et al., 1998; Hanage et al., 2006). MLST approach resolve the issue of inter and intraspecific recombination by concatenating housekeeping genes and minimizing the chances for distorted interpretation of clonal evolution. Housekeeping genes constitute the core genome shared by all strains of a given species and are considered less likely to be a recombinant gene compared to accessory genes of a given species (Daubin et al., 2002; McInerney et al., 2017), thus require additional care for the taxonomic demarcation of *P. parafulva* species to avoid the wrong taxonomic assignment of isolates from the *P. putida* group.

Another genomic trait within bacterial genomes is the presence of genomic islands (GIs), which are typically acquired via HGT-mechanisms. Genes encoded on GIs can render additional adaptive traits in terms of genomic plasticity driven by exposure to environmental factors, which may lead to evolutionary survival (Tumapa et al., 2008; Melnyk et al., 2019). Overall, GIs are classified into four major categories; PAIs (pathogenic islands) that code for virulence genes, MIs (metabolic islands) possessing genes for secondary metabolites biosynthesis, RIs (resistance islands) which usually code for antibiotics resistance, and SIs (symbiotic islands) genes coding for symbiotic associations with other host species. Specifically, we were able to identify 22 GIs integrated into the OS-1 genome via the Island Viewer pipeline.

## Conclusion

In this study, genome sequencing and comparative genome analyzes facilitated the prediction of several genes engaged in the metabolism of aromatic compounds, bacterial growth, phosphorus, and sulfur metabolism etc. The pan-genome analysis highlighted its unique diverse metabolic functions and reveals that OS-1 is equipped with a distinct metabolic profile from the rest of the *P. parafulva* strains. Meanwhile, the strain also showed the presence of various genes responsible for multiple metals and osmotic stress resistance, which provided the genomic basis for the strain to adapt to the external complex harmful environment. However, more research is needed to provide a detailed interpretation of the functions and the regulation of these genes. In-depth genome analysis also facilitated the prediction of virulence factors in the environmental isolate *P. parafulva* OS-1 and antimicrobial resistance genes. Future experimental work is required to validate the functions of these candidate genes and to determine the exact pathogenicity using standard cell culture and animal studies. Analysis of the metabolic potential of OS-1 showed that it contained several gene related to lignocellulose degradation, which further open the door to future developments that will enable the better use of *P. parafulva* OS-1 in biotechnological applications.

## Sequence submission

The genome sequence of OS-1 was submitted to NCBI under BioProject PRJNA907201, BioSample SAMN31956130 and SRA SRR22572176.

## Data availability statement

The datasets presented in this study can be found in online repositories. The names of the repository/repositories and accession number(s) can be found in the article/Supplementary material.

## Author contributions

KK, PS, and SD analyzed the results. RS wrote the original draft and supervised the work. All authors contributed to the article and approved the submitted version.

## Funding

The work was supported by the Ramalingswami Re-entry Grant provided by Government of India.

## Conflict of interest

The authors declare that the research was conducted in the absence of any commercial or financial relationships that could be construed as a potential conflict of interest.

## Publisher’s note

All claims expressed in this article are solely those of the authors and do not necessarily represent those of their affiliated organizations, or those of the publisher, the editors and the reviewers. Any product that may be evaluated in this article, or claim that may be made by its manufacturer, is not guaranteed or endorsed by the publisher.

## Footnotes

^1^ http://rdp.cme.msu.edu/seqmatch/seqmatch_intro.jsp

^2^ http://www.ncbi.nlm.nih.gov/BLAST

^3^ http://www.bioinformatics.babraham.ac.uk/projects/fastqc

^4^ doi:http:ftp.ncbi.nih.gov/pub/wolf/COGs/

^5^ http://imedea.uib-csic.es/jspecies/
