# Supplementary material for: In-depth genome and pan-genome analysis of a metal-resistant bacterium Pseudomonas parafulva OS-1
Source: Front Microbiol. 2023 Jun 20;14:1140249. doi: 10.3389/fmicb.2023.1140249 (PMC10318148; doi:10.3389/fmicb.2023.1140249)
Supplement: Supplementary file 1 [file Data_Sheet_1.zip › Suppl. Tables.DOCX]

**Suppl. Table 1. Biochemical characteristic feature of *P. parafulva* OS-1**

**Characteristic (s) Activity Carbohydrate Activity**

Gram reaction - Sodium gluconate -

Catalase + Glycerol +

Indole - Salicin -

MR - Dulcitol -

VP - Inositol +

Amylase - Sorbitol -

Lipase + Mannitol -

Pectinase - Adonitol -

Catalase + Inulin +

Cellulase + Arabitol -

Temp. tolerance (°C) 45 Erythritol -

Salt tolerance (%) 8% Citrate +

pH 4-10 α-Methyl-D-glucoside -

Swimming + L-Arabinose +

Swarming + Rhamnose +

Twiching + Cellobiose +

**Carbohydrate Activity** Melezitose +

Lactose + α-Methyl-D-mannoside -

Xylose + Xylitol -

Maltose + ONPG -

Fructose + Esculin hydrolysis +

Dextrose - Mannose +

Sucrose + D-Arabinose -

Galactose + Malonate utilization +

Raffinose _+_ Trehalose +

**Suppl. Table 2 Antibiotic sensitivity test of *P. parafulva* OS-1**

| **Antibiotics** |  |
| --- | --- |
|  | **S-33 (ZOI)** |
| Streptomycin (10 mcg) | 15 mm |
| Ampicillin (10 mcg) | 12 mm |
| Gentamicin (10 mcg) | 18 mm |
| Tetracyclin (30 mcg) | 20 mm |
| Kanamycin (30 mcg) | 12 mm |
| Erythromycin (15 mcg) | 20 mm |
| Ciprofloxacine (5 mcg) | 22 mm |
| Fluconazole (25 mcg) | 15 mm |
| Vancomicin (30 mcg) | 25 mm |
| Voriconazole (25 mcg) | 22 mm |

**Suppl. Table 3 The distribution of various CAZymes in OS-1 genomes**

| **ORGANISM** | **GH** | **GT** | **CE** | **AA** | **CBM** | **PL** |  |
| --- | --- | --- | --- | --- | --- | --- | --- |
| *Pseudomonas parafulva* OS-1 | 37 | 37 | 4 | 11 | 4 | 0 |  |
| *Pseudomonas parafulva* DSM 17004 | 5 | 6 | 0 | 2 | 0 | 0 |  |
| *Pseudomonas parafulva* CRS01-1 | 28 | 32 | 4 | 6 | 2 | 3 |  |
| *Pseudomonas parafulva* JBCS 1880 | 30 | 33 | 4 | 5 | 2 | 3 |  |
| *Pseudomonas parafulva* PRS09-11288 | 21 | 33 | 4 | 10 | 1 | 0 |  |
| *Pseudomonas parafulva* NBRC 16636 | 5 | 6 | 0 | 2 | 0 | 0 |  |
| *Pseudomonas parafulva* DTSP2 | 3 | 6 | 0 | 1 | 0 | 0 |  |
| *Pseudomonas parafulva* NS212 | 0 | 6 | 0 | 2 | 0 | 0 |  |
| *Pseudomonas parafulva* NS96 | 1 | 0 | 1 | 0 | 0 | 0 |  |
| *Pseudomonas parafulva* PSB00030 | 0 | 2 | 1 | 0 | 0 | 0 |  |

**Suppl. Table 4 The distribution of metal resistance genes in OS-1 genome**

**Genes Functions**

CzcD Cobalt-zinc-cadmium resistance protein

ZitB Zinc transporter ZitB

CzcA Cobalt-zinc-cadmium resistance protein CzcA

CzcC Heavy metal RND efflux outer membrane protein, CzcC family

CzrB Zn(II) and Co(II) transmembrane diffusion facilitator

CusB/CzsB Putative copper efflux system protein CusB

CusR Copper-sensing two-component system regulator CusR

CusS Copper sensory histidine kinase CusS

CutF Copper homeostasis protein CutF

CutE Copper homeostasis protein CutE

SEP Putative silver efflux pump

CusB/CzsB Probable Co/Zn/Cd efflux system membrane fusion protein

NccA Nickel-cobalt-cadmium resistance protein NccA

NccB Nickel-cobalt-cadmium resistance protein NccB

CRA Cadmium-transporting ATPase

arsA Arsenical pump-driving ATPase

arsB Arsenic efflux pump protein

arsC Arsenate reductase

arsH Arsenic resistance protein ArsH

CorC Magnesium and cobalt efflux protein CorC
