# Supplementary figures and images for: In-depth genome and pan-genome analysis of a metal-resistant bacterium Pseudomonas parafulva OS-1
Source: Front Microbiol. 2023 Jun 20;14:1140249. doi: 10.3389/fmicb.2023.1140249 (PMC10318148; doi:10.3389/fmicb.2023.1140249)

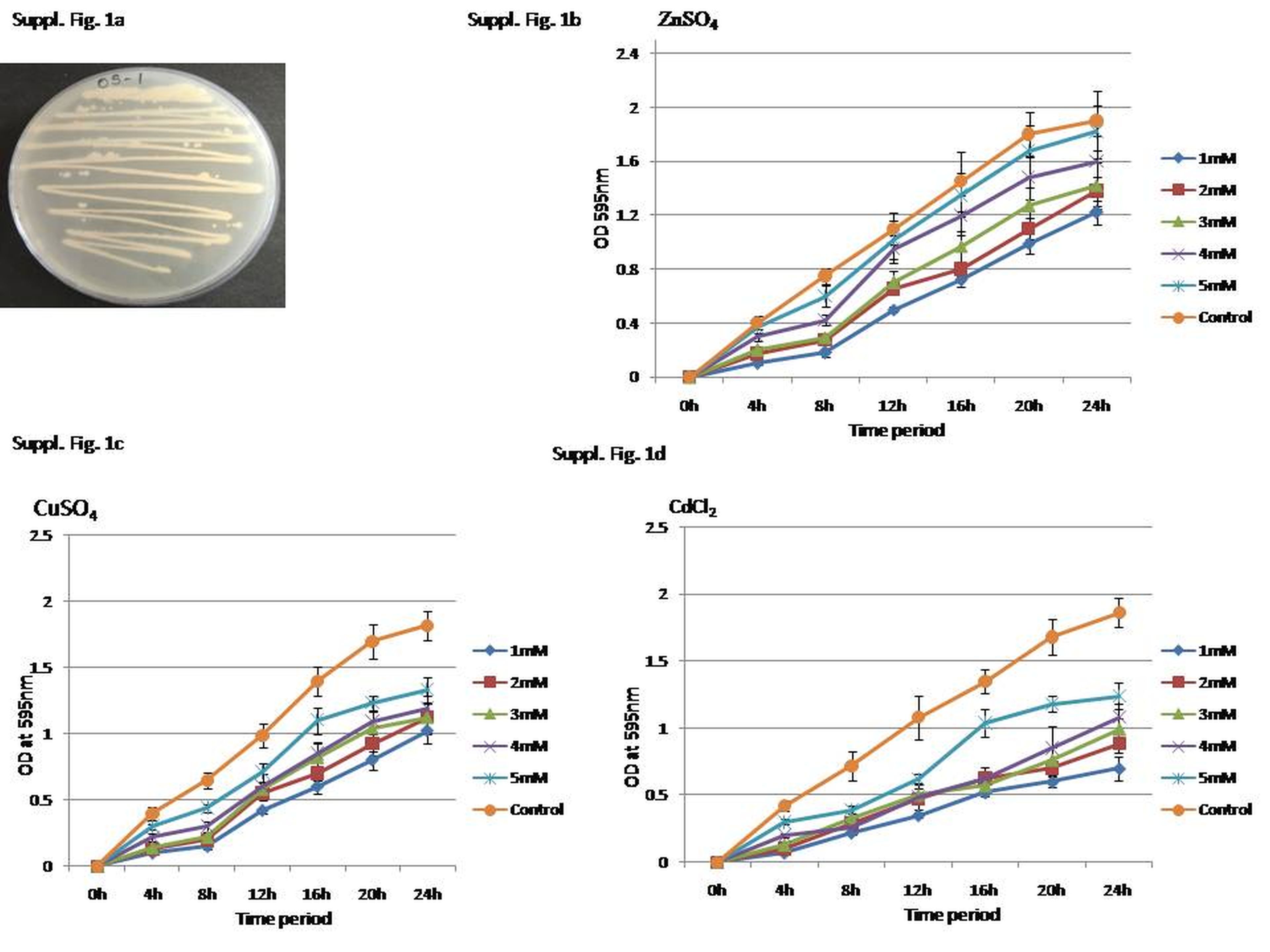

Supplement: Supplementary file 1 [file Data_Sheet_1.zip › Figure_S1_a-d.JPEG]

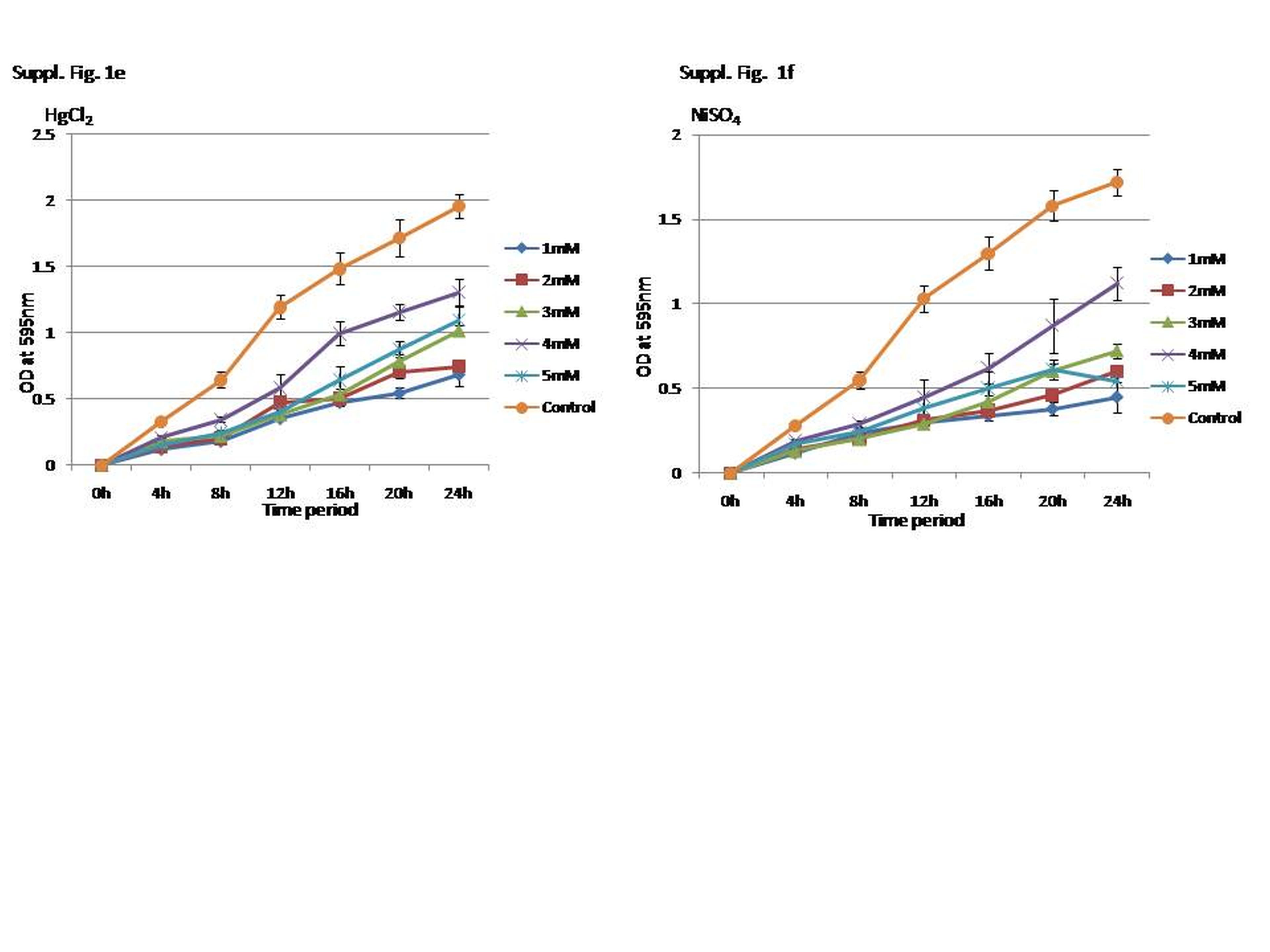

Supplement: Supplementary file 1 [file Data_Sheet_1.zip › Figure_S1_e-f.JPEG]

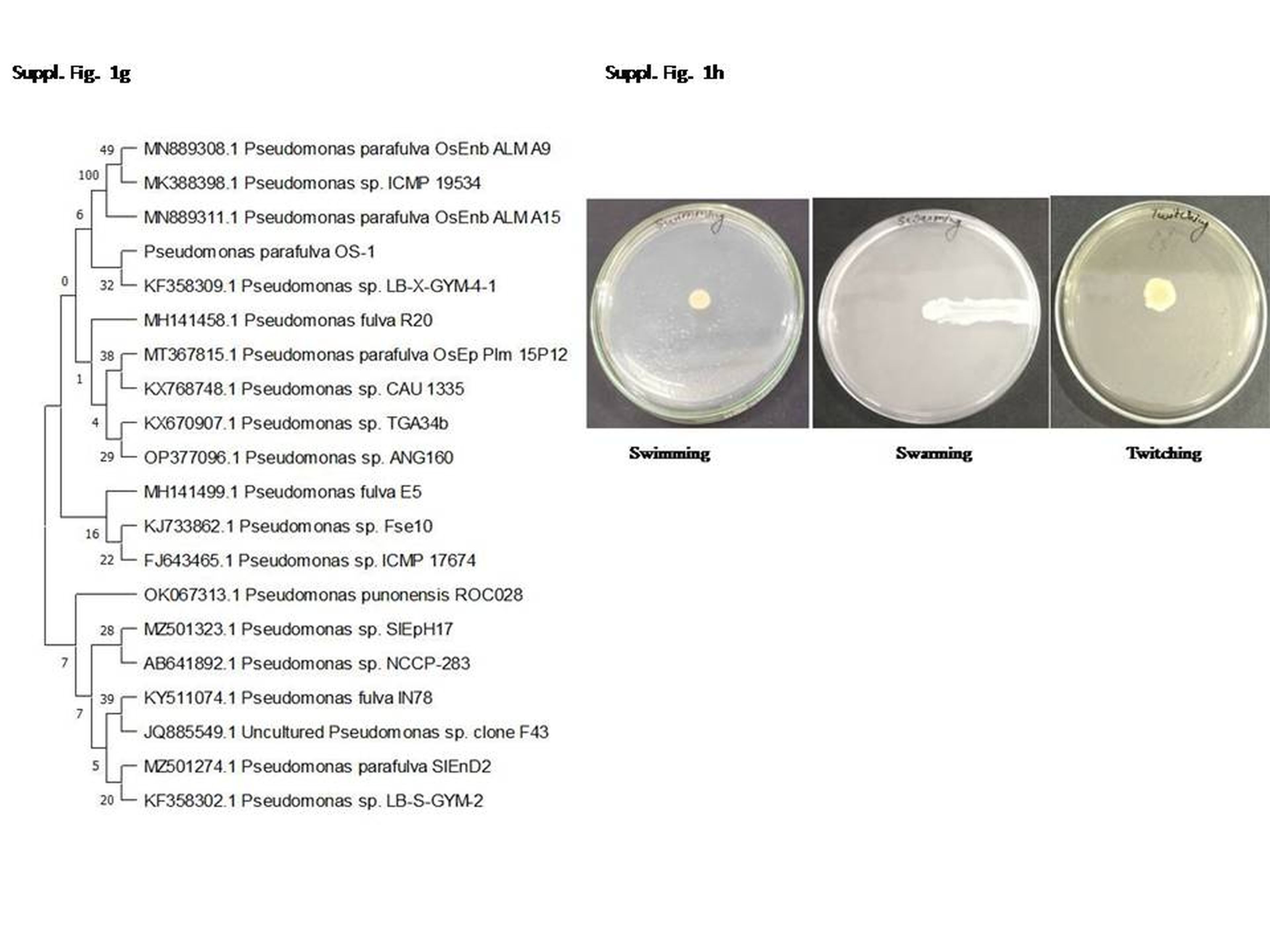

Supplement: Supplementary file 1 [file Data_Sheet_1.zip › Figure_S1_g-h.JPEG]

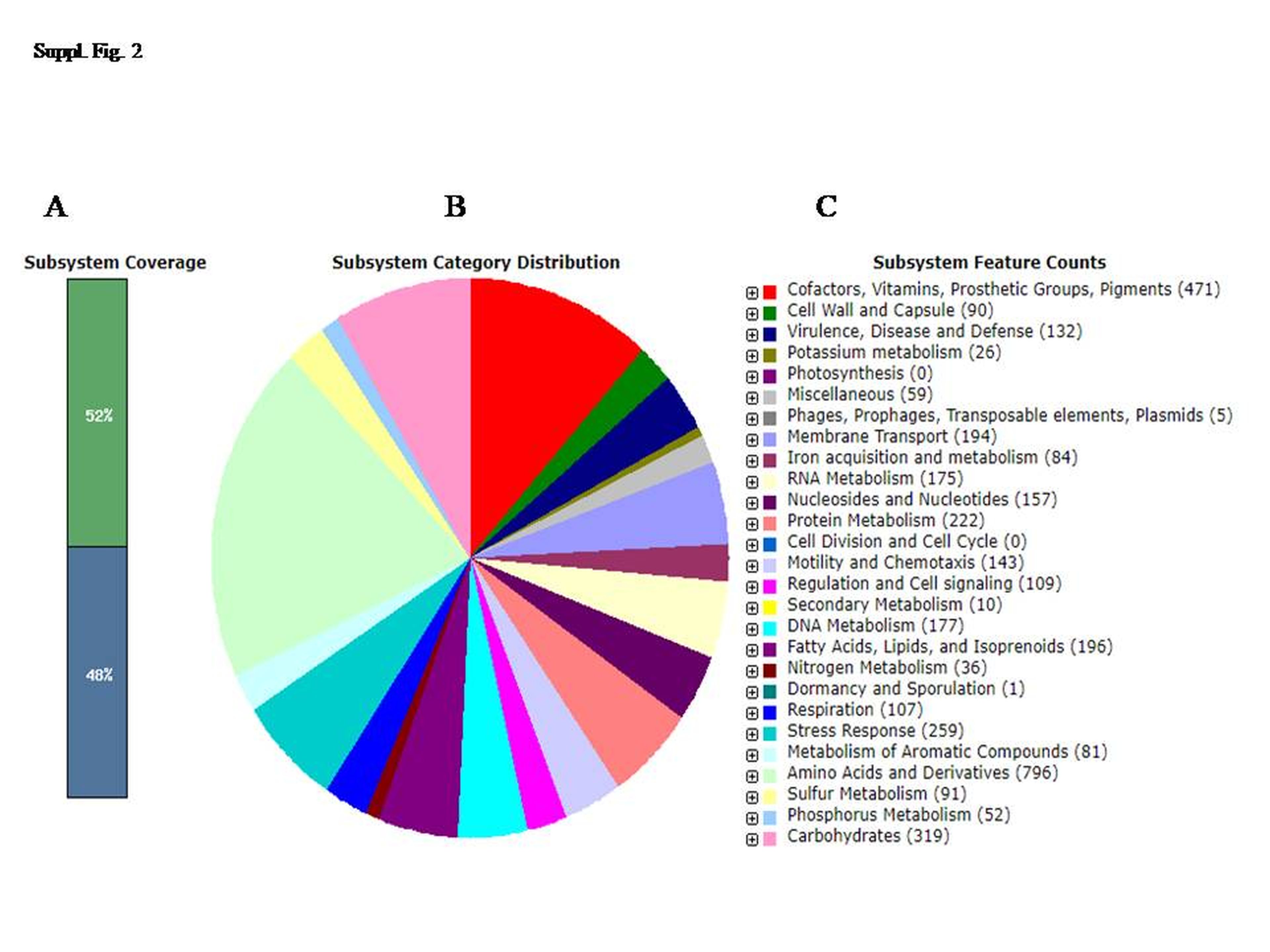

Supplement: Supplementary file 1 [file Data_Sheet_1.zip › Figure_S2.JPEG]

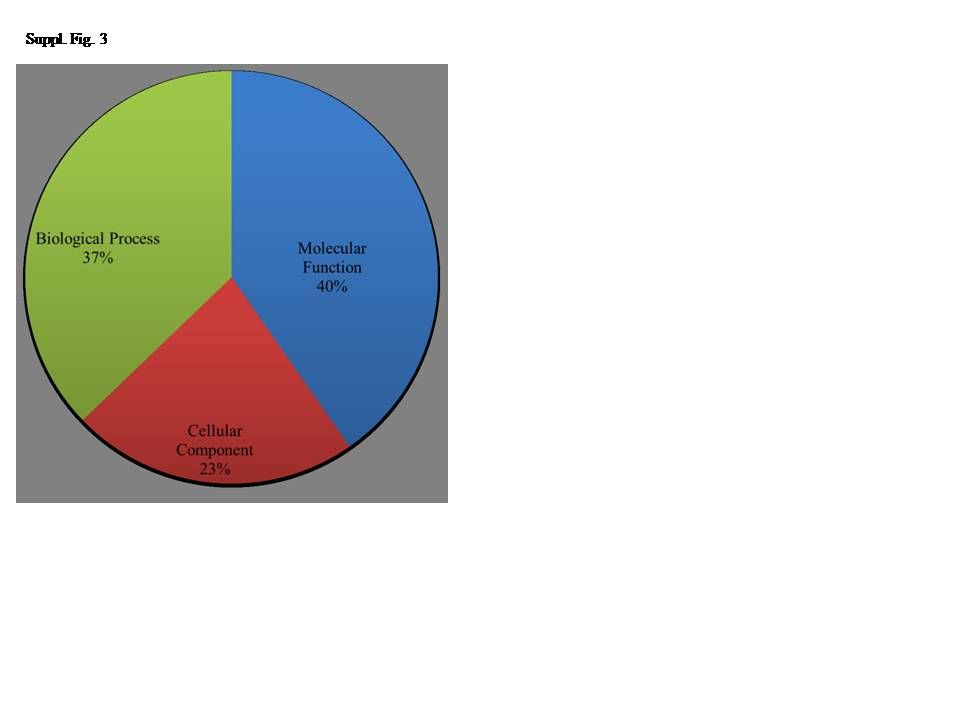

Supplement: Supplementary file 1 [file Data_Sheet_1.zip › Figure_S3.JPEG]

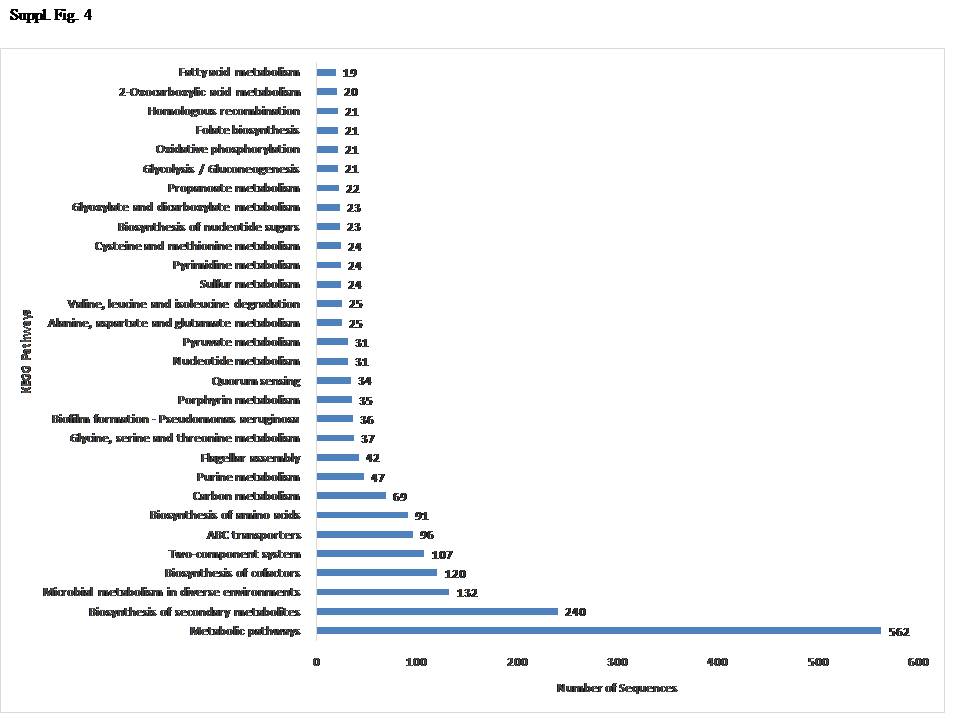

Supplement: Supplementary file 1 [file Data_Sheet_1.zip › Figure_S4.JPEG]

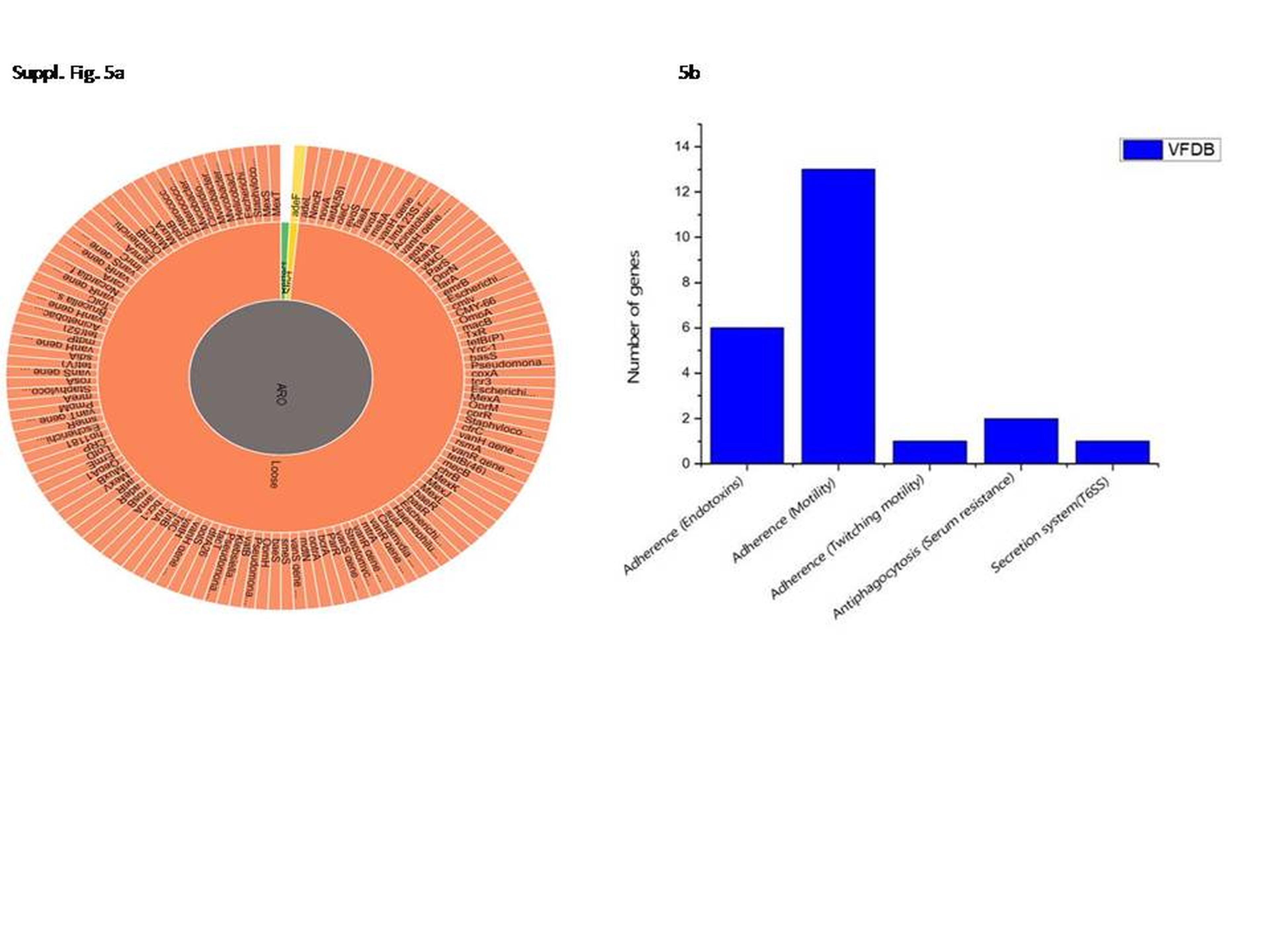

Supplement: Supplementary file 1 [file Data_Sheet_1.zip › Figure_S5_a-b.JPEG]

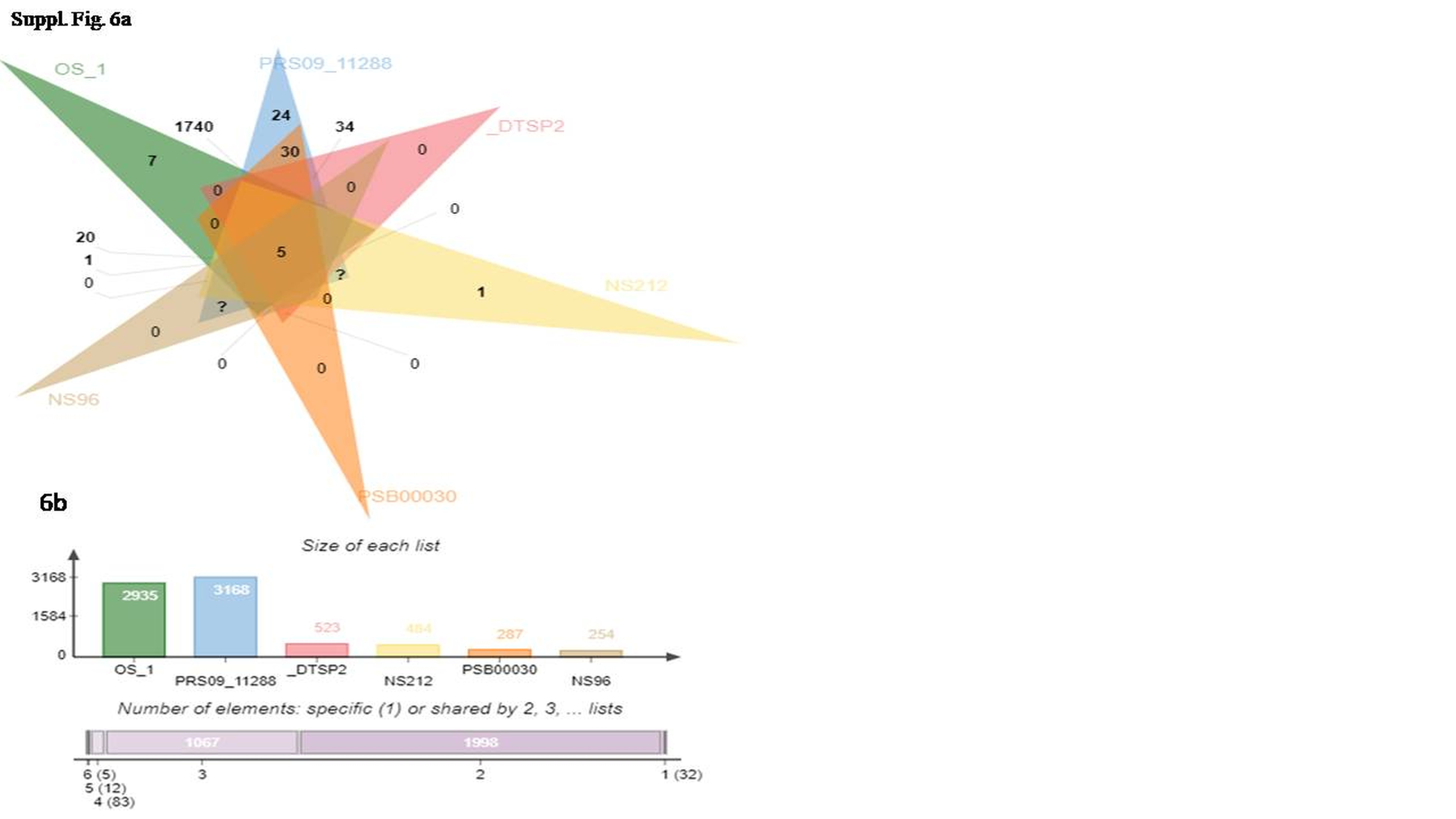

Supplement: Supplementary file 1 [file Data_Sheet_1.zip › Figure_S6_a-b.JPEG]

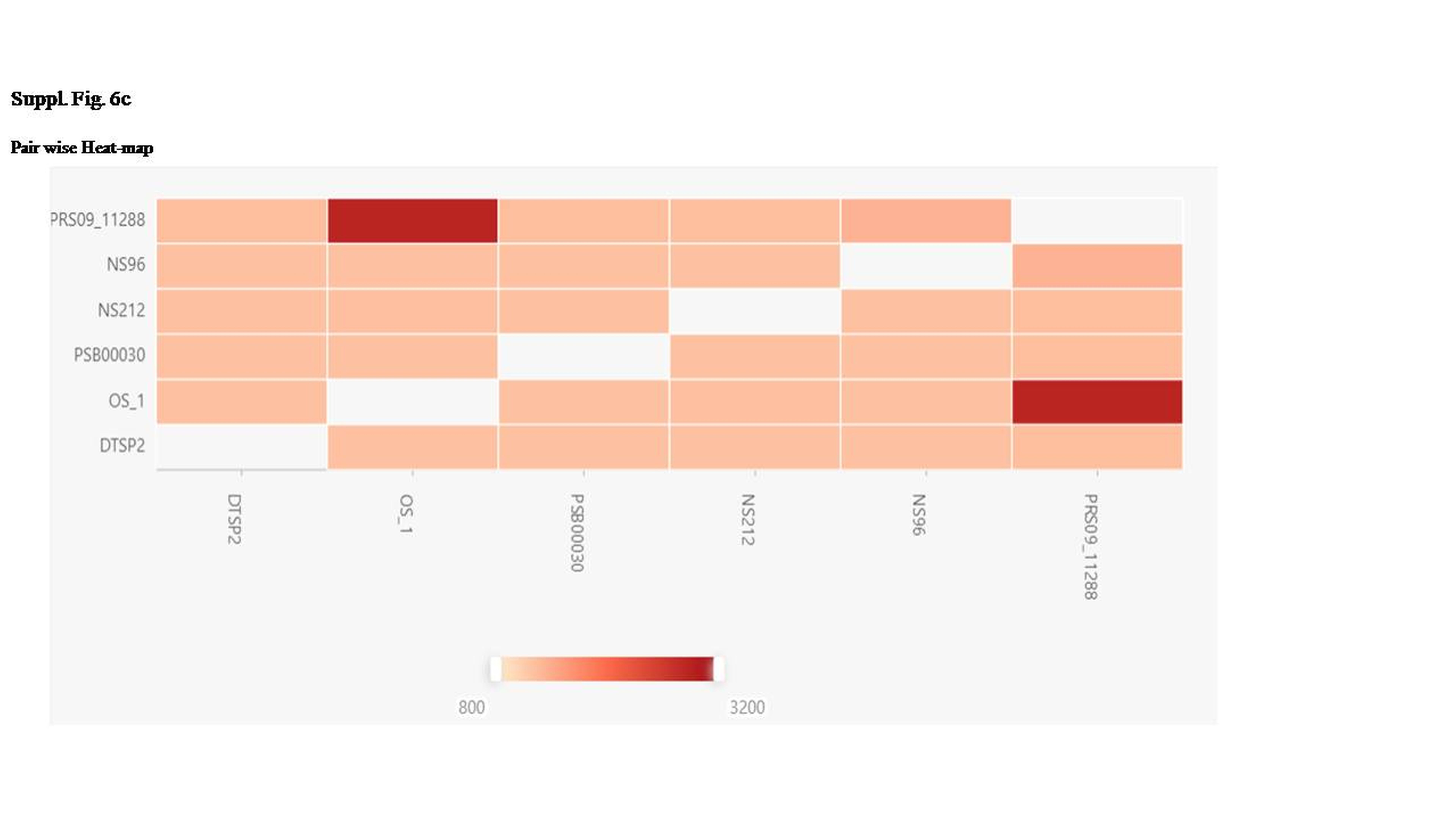

Supplement: Supplementary file 1 [file Data_Sheet_1.zip › Figure_S6_c.JPEG]

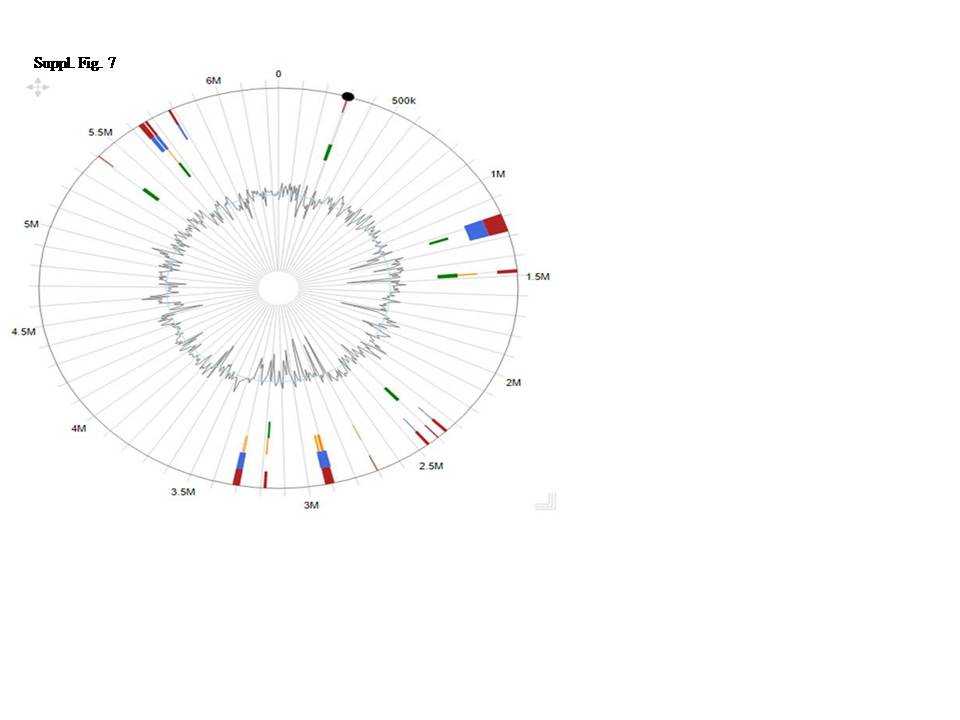

Supplement: Supplementary file 1 [file Data_Sheet_1.zip › Figure_S7.JPEG]

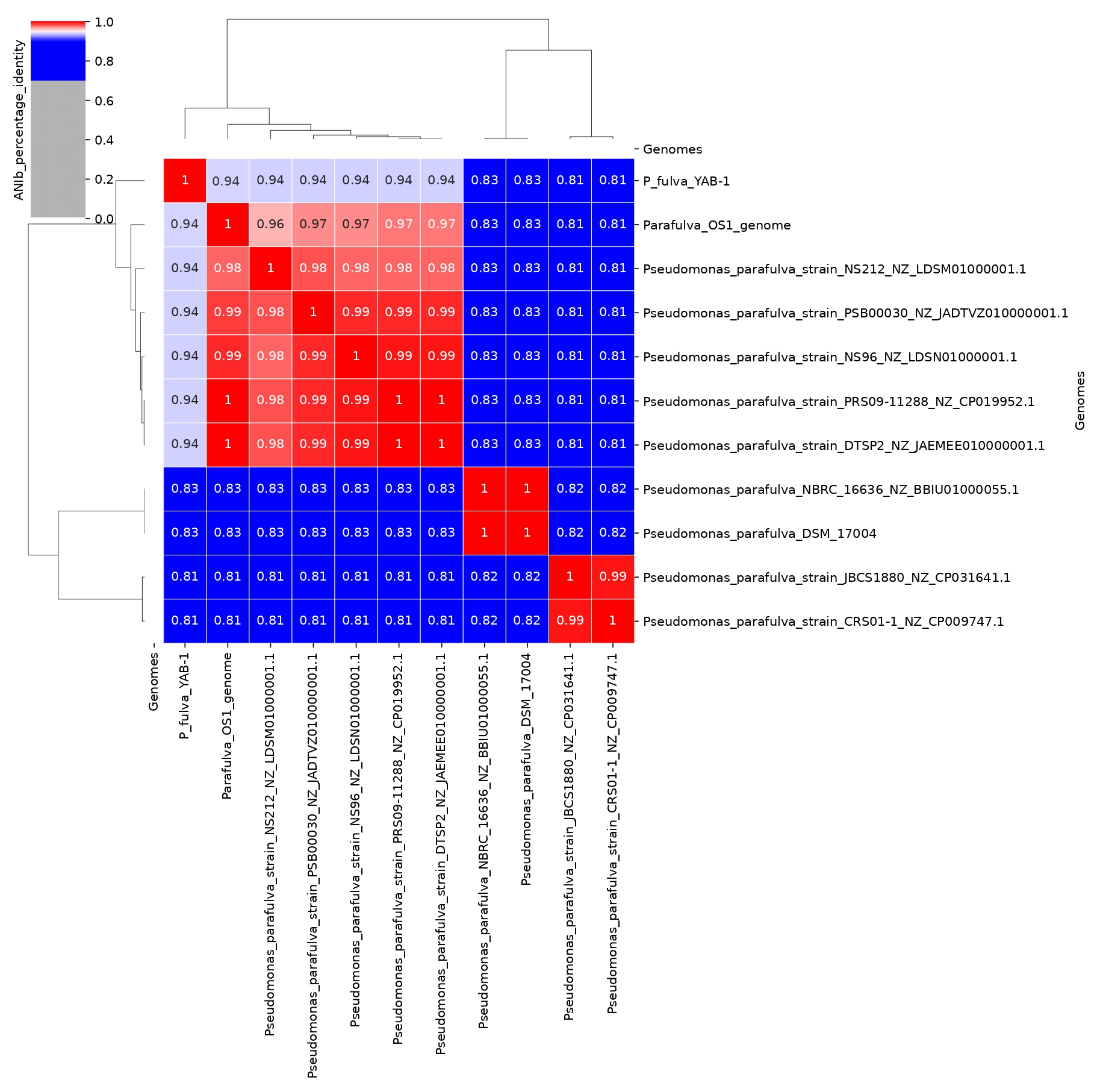

Supplement: Supplementary file 1 [file Data_Sheet_1.zip › Figure_S8.JPEG]
